# Refining Electronic Tagging of Marine Animals: Computational Fluid Dynamics and Pelagic Sharks

**DOI:** 10.3390/ani15202956

**Published:** 2025-10-13

**Authors:** Tobias C. Maillard, Francesco Garzon, Lucy A. Hawkes, Gavin R. Tabor, Matthew J. Witt

**Affiliations:** 1 Engineering Group, Faculty of Environment, Science and Economy, Streatham Campus, University of Exeter, Exeter EX4 4QF, UKg.r.tabor@exeter.ac.uk (G.R.T.); 2 Biosciences, Hatherly Laboratories, Faculty of Health and Life Sciences, Streatham Campus, University of Exeter, Exeter EX4 4PS, UK; fg350@exeter.ac.uk (F.G.); l.hawkes@exeter.ac.uk (L.A.H.)

**Keywords:** computational fluid dynamics (CFD), animal-borne data loggers, shark tagging, mako shark, tag impact, drag coefficient, biologging

## Abstract

**Simple Summary:**

Scientists commonly attach electronic tags to marine animals to study their movements and behavior in the ocean. However, these tags may have consequences by making it harder for animals to swim efficiently, potentially harming their recovery and survival while also affecting the quality of research data collected. This study used water flow simulations to understand how different types of tags and attachment locations affect the swimming ability of mako sharks of various sizes. The research tested multiple swimming speeds and tag configurations to measure changes in water resistance, which makes swimming more energy intensive. Results demonstrated that tags attached to fins significantly increased drag up to ~30%, making swimming more demanding. Tags attached to the main body performed better, causing minimal problems for larger sharks over 1.5 m long, but still created notable issues for smaller sharks, requiring about 7% more daily energy. Based on these findings, researchers recommend size-based limits for tagging and provide specific guidelines for optimal tag placement. This work helps scientists make better decisions about animal tagging practices, ensuring research can continue while minimizing harm to marine wildlife and maintaining reliable scientific data.

**Abstract:**

Animal-borne tags are widely used for tracking and monitoring the movements, behaviour, and ecology of marine animals. Tagging can, however, adversely affect the hydrodynamic force balance and welfare of tagged animals, and consequently, the reliability and accuracy of data, such as by increasing drag, altering swimming characteristics, and reducing the survival rate of tagged animals. Therefore, it is important to understand and quantify the impact of tagging on marine animal hydrodynamics and to optimize the choice of tag and attachment position. In this study, computational fluid dynamics (CFD) modelling is used to simulate the flow around tagged and untagged mako sharks (Lamnidae) across their swim speed range for two dominant tag shapes, tagging sites, and body sizes. The results indicate that fin mounted tags can have a significant impact on shark hydrodynamics and energetic balance, increasing drag between 17.6% and 31.2% for a mako shark (2.95 m fork length) across the range of flow velocities tested (0.5 to 9.1 m/s). In comparison, the optimal tagging site for archival tags attached to the dorsal musculature leads to a minimal increase in drag for the larger sharks (>1.5 m), which becomes considerable for small sharks (1 m fork length; 5.1% to 7.6% increase) and leads to an average energetic cost equivalent to 7% of the daily energetic requirement of an untagged animal. Other aspects of the force balance are considered, which reveal a range of varied and complex effects. Recommendations for animal size thresholds (>1.5 m FL) and refinements of tagging practice are suggested.

## 1. Introduction

Marine megafauna such as sharks, sea turtles, cetaceans, and pinnipeds play diverse and critical roles in maintaining marine ecosystem structure and function. To provide effective management of their populations and the ecosystems they inhabit, it is necessary to understand their behaviour, ecology, and population dynamics. These are especially important when considering the growing threats from anthropogenic activities, including habitat destruction, overfishing, climate change, and pollution [1,2,3]. Studying these animals in their natural habitats often poses several challenges, such as the logistical and ethical limitations of performing field research [4,5]. One widely used approach to overcome this is the use of animal-borne data loggers (“tags”) attached to marine animals to record information such as location, depth, speed and, or acceleration of the animal [2].

Tagging does, however, present some potential drawbacks [2,4,6], particularly the impact of the tag on the host animal [7,8,9,10]. Inappropriately shaped, sized or poorly positioned tags may affect their natural behaviour, reduce the value of collected data, and potentially affect their energy expenditure, welfare, and survival [11,12,13,14]. These impacts depend on a wide range of factors such as the size, shape and effective weight of the tag [15], the attachment position and method on the animal [16], the size, shape and locomotory mechanisms of the animal [17], the range of speeds and distances covered by the animal [18], and the flow characteristics and properties of the fluid in which the animal is moving. Nevertheless, it is necessary to assess and minimize impacts that tagging has on the animal, both to ensure the validity and reliability of the data, and to understand, quantify, and reduce any adverse effects imparted on the animal through tagging [14,19]. In avian biologging, a commonly accepted rule is that tags should weigh no more than 3% of the host body mass [20]; similarly, a 2% rule has been adopted in teleost biologging [21,22,23], although perhaps not as strictly as in the avian world. The 2% rule is based on the estimation of how much additional weight derived from the tag that the teleost swim bladder may compensate for, but may not account for the buoyancy of the tag and is sometimes erroneously applied to the weight in air of the tag, rather than its weight in water. Furthermore, this rule is not applicable to marine animals that do not possess a swim bladder, nor does it account for the other hydrodynamic effects that the tag may create when attached externally to the body, such as increased drag and lift, which vary with swimming speed. As such, the 2% rule is not universally applicable and has received criticism for its lack of empirical support [21,24]. Changes in drag and other hydrodynamic forces caused by external tags are known to have consequences on the behaviour, physical form and energy balance of marine animals (e.g., [25,26,27,28]) and should be considered when determining ethical thresholds for the field.

One method that has been used to assess the hydrodynamic impact of tagging is Computational Fluid Dynamics (CFD) [2,7,8,10,18]. CFD is a numerical technique that solves the governing equations of fluid flow using discretization schemes and iterative algorithms, allowing for complex flows around arbitrary geometries to be simulated with high accuracy and resolution. CFD can provide detailed information on the pressure and shear stress distributions on the surface of a marine animal and tag, as well as the flow field around it. These data can, in turn, be used to calculate and compare the hydrodynamic forces acting on different tag shapes or attachment sites for different animal geometries or flow conditions. A limited number of studies have used CFD to model the additional cost of carrying biologging tags for marine animals including penguins [11,12,17], marine turtles [16], seals [8,10,29] and cetaceans [7,15,30]. These studies indicate that the cost of transport may increase by as much as 149% [16] depending on the tag size, configuration and animal shape and speed, and animals may reduce their travel speed or locomotion style to compensate [12,17,29].

Sharks are amongst the most commonly tracked marine taxa [31] and the number of studies that have deployed electronic tags on sharks has increased three-fold in the last decade (2010–2020) [32]. The vast majority of tracking studies focused on sharks use externally mounted tags, often attached to the dorsal fin or to the dorsal musculature [32]. While tracking of sharks has been successful and has infrequently resulted in serious injury or mortality in study animals, tag attachment has been found to cause behavioural alterations and sub-lethal physiological impacts [19,32,33,34,35,36]. The impact of tags is likely influenced by factors such as attachment site, size and shape, buoyancy, anchoring method, animal physiology and behaviour, and environmental conditions [26,36,37,38,39]. As the field continues to expand, researchers should seek to better understand how electronic tags may influence study animals and how to refine tags and tagging techniques to minimise impact on sharks. To this end, here we use CFD to investigate the hydrodynamic impact of commonly used biologging tags on mako shark (*Isurus oxyrinchus*), which is broadly representative of the streamlined pelagic lamniform body plan, to explore strategies for minimizing hydrodynamic impact and improving tag design. Specifically, we ask: (1) How do tag shape and placement affect hydrodynamic drag on mako sharks? (2) Can CFD modelling inform ethical thresholds for tag design in pelagic sharks?

## 2. Materials and Methods

Computational Fluid Dynamics (CFD) is the process of calculating the fluid flow around a given shape (“geometry”) through the numerical solution of the physical laws governing the conservation of mass and momentum, as expressed by the Navier-Stokes equations. CFD results approximate real-world flows, with the continuum fields of pressure and velocity being represented computationally by values at a finite number of points in space. This process of discretisation is typically carried out by use of the Finite Volume method, where the computational domain is split into numerous (often millions) of small polyhedral volumes referred to as cells or control volumes. These cells are non-overlapping and tightly packed, forming a mesh, with the dependent variables being associated with the cell centre locations. The continuum equations governing the flow can be discretised on this mesh to form a set of difference equations that can be solved numerically through iterative matrix solvers. For more complex systems the Navier-Stokes equations may require supplementary equations representing additional physics. Many real-world flows involve the presence of turbulence. Turbulent flows exhibit complex flow structures across a wide range of spatial and temporal scales, which are typically too costly to simulate completely. Instead, we rely on a process of turbulence modelling, in which the Navier-Stokes equations are averaged (to form the Reynolds Averaged Navier Stokes or RANS equations) and solved for the average velocity and pressure, together with additional quantities such as turbulent kinetic energy, which describe the stochastic component of the turbulent flow in a statistical manner. A variety of RANS turbulence models are available; commonly used are the k-e and k-w-SST models, used in this work.

Applying CFD methods to resolving flow around a marine animal typically involves the following steps: (i) determining a virtual bounding box containing a simplified geometry of the animal/tag; (ii) meshing, which is the discretization of the computational domain into a finite number (usually thousands or millions) of volumes (“cells”) of arbitrary shape and size, which will constitute the units to which calculations are applied; (iii) setting of boundary conditions (i.e., how the walls of the geometry and the computational domain are to interact with the flow) and key physical parameters (water flow velocity, turbulence intensity, fluid density); (iv) modelling, in which the equations are used to compute flow variables for each finite volume in an iterative process, continuing until estimates converge towards a solution; and (v) postprocessing and analysis of the calculated results.

CFD simulations were performed using OpenFOAM (OpenFOAM10, Foundation version) [40], an open-source software that provides a wide-ranging set of tools for mesh generation, numerical solving and post-processing utilities. The simulation uses the simpleFoam steady-state solver with bounded second-order accurate spatial discretization for the gradient terms, and upwind differencing for the transport terms for stability. Diffusion terms are computed accurately even on irregular meshes. Pressure is solved efficiently using a multigrid approach, while velocity and turbulence quantities use iterative Gauss–Seidel-based solvers. Residual convergence tolerance was set to be 5.5 × 10^−4^ for the pressure equation, 5 × 10^−4^ for the velocity and turbulence quantities.

### 2.1. Preparation of Geometries

#### 2.1.1. Mako Shark Model

**Figure 1:**
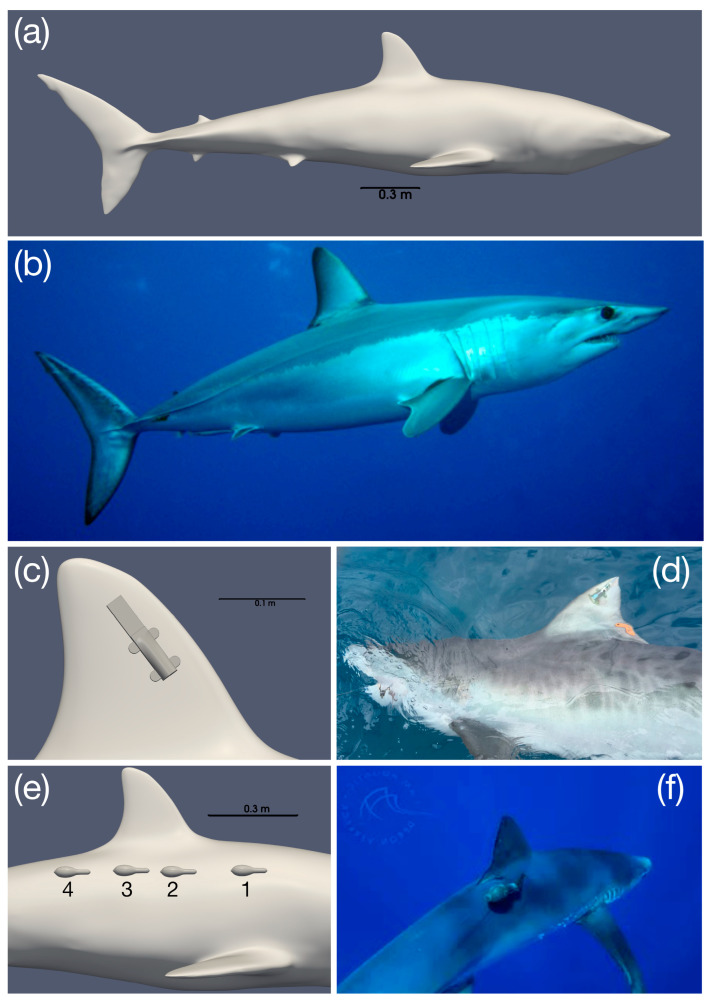
CAD models of shark and tag types used for modelling. (**a**) 3D CAD model of a mako shark and accompanying photo of a mako shark (**b**); (**c**) lateral view of SPOT-258 tag attached to a mako shark dorsal fin (3D model) with accompanying photo of tag deployed on a shark ((**d**); photo credit: Rachel Graham, MarAlliance); and (**e**) lateral view of mako shark with four tag attachment sites (left; 1–4) for a MiniPAT and photo of exemplar tag attachment on a shark ((**f**); photo credit: Deron Verbeck and Melanie Hutchinson).

A 3D digital model of a mako shark was sourced from the online open-access database Digital Life (http://digitallife3d.org, accessed on 11 April 2024). The model was imported into Blender (Blender Foundation, 2023, version 3.5), an open-source 3D creation software, where it was modified by removing eyes, teeth, and gills, and closing the mouth (Figure 1a), and smoothed to simplify the shape and improve the suitability for CFD analysis. Further geometry checks were conducted in ANSYS Design modeller (Ansys Inc., ANSYS Workbench version 2021 R1) to eliminate erroneous cells and ensure the model was robust (e.g., surfaces join without holes or overlap, or other malformations that can occur).

In fluid mechanics, significant characteristics of the flow are determined by the Reynolds number (Equation (1)), which is a dimensionless parameter formed from characteristic length and velocity scales of the problem. This characterises the ratio of inertial to viscous forces and determines whether the flow is laminar or turbulent.

(1) $$Re=\frac{\rho \cdot U_{\infty }\cdot L}{µ}$$

where ρ is the density of the fluid (kg/m^3^), $U_{\infty }$ is the freestream velocity (m/s), L is the characteristic length (m), μ is the dynamic viscosity (kg/m.s). The characteristic length, *L*, was taken to be the fork length of the animal (i.e., the distance from nose to fork in the caudal fin).

Characteristic length and maximum frontal cross-sectional area of the shark were measured to calculate dimensionless force coefficients and other parameters (e.g., Reynolds number, etc.). The mako model origin was set to its geometric centre of mass and then exported for use in OpenFOAM. The mako model represented an adult life stage and had a fork length of 2.95 m [41]. To represent younger age classes, the model was scaled using the Blender transform function with a uniform scaling factor to achieve fork lengths of 1, 1.5, 2, and 2.5 m. Reynolds number similarity was preserved across scaled models.

#### 2.1.2. Tag Model and Tagging Site

Of the shark tracking studies assessed by [32], 76% used Pop-up Archival Transmitting (PSAT) tags and 34% used fin-mounted Smart Position Or Temperature (SPOT) tags. As such, two tag shapes were modelled in this study: Wildlife Computers (i) MiniPAT and (ii) SPOT-258 fin-mount tag. Wildlife computers (Washington, DC, USA) is a producer of tracking technologies and amongst the most widely used in the sector [32,33].

The MiniPAT (model MiniPAT-390) model was obtained as an STL file from Wildlife Computers, while the SPOT-258 tag was re-created in SolidWorks (SolidWorks Corporation; version Solidworks 2021), based on the measurements of its physical geometry. In both cases, the antenna was removed to simplify the geometry and make it suitable for CFD analysis. The tags were “attached” to the shark geometries in Blender, placing them in their respective positions, along the body of the shark for the MiniPAT, and on the dorsal fin for the SPOT tag (Figure 1). Due to the curvature of the MiniPAT and the sensitivity of CFD analysis to small cavities in the geometry, this tag was slightly indented into the shark geometry to eliminate gaps between the two geometries that are unlikely to significantly alter the flow characteristics but could lead to modelling issues.

### 2.2. Meshing

#### 2.2.1. Base Mesh

The modelling domain was a rectangular volume (16 × 8 × 8 m) with the centre of the shark at the origin. An initial background mesh of hexahedral cells was created using “blockMesh” in OpenFOAM that can create simple meshes. The modelling domain was broken into eight blocks split symmetrically about the origin, and grading factors were applied to increase mesh refinement. Inlet and outlet boundary patches were created, as well as identifiers for the unused domain walls.

#### 2.2.2. Mesh Refinement

“SnappyHexMesh” (SHM) was used to refine the base mesh and improve resolution of the cells. SHM is an OpenFOAM utility that can create high quality unstructured meshes dominated by hexahedral cells from triangulated surface geometries, and consists of three main steps: castellation, snapping, and layer addition. This study only used the first two steps, as layer addition was not necessary for the wall model-based approach given the sufficient surface refinement. The castellation step creates a mesh of hexahedral cells that fit the geometry by splitting and removing cells that intersect with the surface. The snapping step moves the vertices of the castellated mesh towards the geometry surface to improve the alignment and accuracy of the mesh and modifies the cell connectivity and topology to avoid overlapping or distorted cells. The meshing process used a heuristic optimization strategy inspired by genetic algorithms. This approach involved determining mesh settings based on initial research [42,43,44], then running batches of meshing, and selecting only the most successful original meshes to be ‘parent’ meshes, where parameter variations were applied to the mesh settings (based on the knowledge of what each setting should do) to iterate and improve mesh quality until acceptable meshing was achieved.

#### 2.2.3. Mesh Quality Inspection

The quality of the generated mesh was evaluated using “checkMesh”. Erroneous mesh properties were inspected using “ParaView” (version 5.10.1) in OpenFOAM to identify, inspect, and remove geometric anomalies that could compromise simulation stability. Final meshes were selected based on key mesh indicators, including having cell faces aligned with expected flow direction, ensuring the shark surface closely matched the mesh with uniform boundary layer resolution, and that most cell refinement focused on the shark. Further details of mesh generation and refinement can be found in Supplementary File S1A,B.

### 2.3. Modelling Framework

#### 2.3.1. Numerical Method

The flow around the shark was modelled using the “simpleFoam” solver in OpenFOAM, which is based on a finite volume method and a pressure-velocity coupling algorithm. The solver applies the simplified form of the Navier-Stokes equations for a constant density (incompressible) fluid with a time-independent (steady) flow through each cell created during meshing, and uses interpolation schemes to approximate the flux of water across the faces of the volumes while ensuring that the laws of mass and momentum are satisfied locally and globally [45,46]. A number of key assumptions were made to define the modelling conditions: (i) the shark was assumed to have a rigid body with no active propulsion (i.e., no movement of fins of tail was modelled), so that the drag force represents the thrust required for forward motion; (ii) the effects of gravity and buoyancy were neglected, due to the similar density of the shark to saltwater [47,48]; and (iii) flow speeds were assumed to be sufficiently high to justify the use of a turbulence model, although at lower flow speed some laminar and transitional boundary layer behaviour may occur.

#### 2.3.2. Turbulence Modelling

Turbulence is a phenomenon that occurs when a fluid flow exhibits chaotic and multi-scale structures due to instabilities and fluctuations. Turbulence is difficult to model accurately because it involves a wide range of length and time scales, which are not computationally feasible to resolve by direct numerical simulation for most cases. Therefore, different types of turbulence models are employed, which are mathematical formulations that attempt to capture the effects of turbulence on the mean flow variables, such as velocity and pressure.

Reynolds-averaged Navier-Stokes (RANS) modelling is a widely used approach for simulating turbulent flows. The most widely used and validated turbulence models in engineering applications are the k-epsilon (k-ε) model and the k-omega shear stress transport (k-ω-SST) model [46,49,50]. The SST k-ω model is generally more robust and accurate; however, it is sensitive to initial boundary conditions and requires a larger computational resource than simpler models. Therefore, k-ε models were run first and their solutions were then used to initiate SST k-ω model, achieving faster and more reliable convergence.

#### 2.3.3. Near-Wall Modelling

The behaviour of the flow near walls (i.e., close to the shark/tag body) requires particular attention in modelling, as the flow tends to turn from turbulent to laminar in proximity of the wall. To resolve the flow at the shark surface (walls), the wall function approach was adopted. The wall function approach applies empirical derived equations to approximate the behaviour of the viscous sublayer and buffer region, requiring a y+ value (a metric related to the height of the mesh cells nearest the wall) range of 30–300 (first cell centre located within the logarithmic layer), providing a significant reduction in cell count and computational resource compared to other widely applied methods [51].

#### 2.3.4. Physical Parameters

Mako sharks typically inhabit water temperatures in the range of 17 to 22 °C [52], therefore the properties of seawater at 20 °C were adopted for this study, giving a density (ρ), of 1025 kg/m^3^ and a dynamic viscosity (μ) of 0.00109 N.s/m^2^. Water flow velocity was set to resemble the typical range of swimming speeds of the mako shark, which were obtained from published literature, and the flow regimes were matched to these speeds (Table 1). The Reynold’s number was calculated for each speed according to Equation (1), where $U_{\infty }$ is the uniform free stream velocity (m/s).

The initial field values of k, epsilon, and omega, as well as the fixed inlet values for these quantities were calculated to initialise each simulation using the ‘rule of thumb’ equations, where Ti is free-stream turbulent intensity [56].

(2) $$k=\frac{2}{3}{\left(U_{\infty }\cdot T_{i}\right)}^{2}$$

(3) $$\varepsilon =0.09^{3/4}\cdot \frac{k^{3/2}}{0.07L}$$

(4) $$\omega =\frac{\varepsilon }{k}$$

### 2.4. Model Validation

To ensure results of simulations were not influenced by the mesh geometry, a mesh independence study was conducted to determine the optimal mesh resolution for the CFD simulations using the “simpleFoam” solver with the k-ε and k-ω SST turbulence models.

Simulations were run on increasingly refined meshes, using the original shark geometry (*L* = 2.95 m), free stream velocity $U_{\infty }=9.1\;m/s$, and a turbulence intensity of 2%. Net viscous and net pressure forces acting on the shark in the x, y, and z directions were calculated and compared between cases, and mesh refinement was terminated when converged was achieved. The convergence tolerance was set to an initial pressure residual value of 5.5 × 10^−4^.

Finally, a validation study was conducted to verify the accuracy of the CFD simulations against experimental data. The validation case used was the DARPA SUBOFF submarine model with tow tank test results [57,58]. The study was chosen as it exhibits similar geometric characteristics as the mako shark geometry (e.g., long-streamlined body, similar characteristic length and maximum cross-sectional area) and uses stream velocities that generate a similar Reynolds number range as that required by this study. Details of the validation procedure can be found in Supplementary File S1C.

### 2.5. Estimation of Drag, Lift, and Pitch Momentum

Forces acting upon the model shark are computed by integrating the pressure force and shear stresses over its surface. The net force can be decomposed into three components: the drag force, *F_D_*, which is parallel to the direction of flow; the lift force, *F_L_*, which is perpendicular to both the flow and the shark body axis, causing an up-or-down movement of the shark’s body; and the side force, *F_S_*, which is perpendicular to both the flow and the lift force. The pitch moment, *M_P_*, is the tendency of a body to rotate about the pitch axis relative to its centre of rotation.

The coefficients of drag *C_D_* lift, *C_L_*, and pitching moment, *C_M_* are defined as:

(5) $$C_{D}=\frac{2\cdot F_{D}}{\rho \cdot A\cdot {U_{\infty }}^{2}}$$

(6) $$C_{L}=\frac{2\cdot F_{L}}{\rho \cdot A\cdot {U_{\infty }}^{2}}$$

(7) $$C_{M}=\frac{2\cdot M_{P}}{\rho \cdot A\cdot L\cdot {U_{\infty }}^{2}}$$

The forces and coefficients are calculated automatically in OpenFOAM by including and adapting the “forces” and “forceCoeffs” function objects from the standard library.

Various post-processing techniques were applied in “ParaView”, to analyse the flow behaviour and characteristics in each scenario, such as flow separation, wake formation, turbulence properties, wall shear stress and pressure distribution, through a variety of vector plots, contour plots, streamlines, and other field value and surface plots. Additionally, graphs of Reynolds number and flow velocity against the forces and force/moment coefficients were plotted for the tagged and untagged cases to compare the overall impacts to the force balance.

### 2.6. Tagging Site Comparison

We investigated the effects of tagging site for two different tag types: SPOT-258 and MiniPAT-390. SPOT-258 tags have only one attachment position on the dorsal fin (Figure 1c). MiniPAT position was evaluated at four potential attachment sites distributed along the dorsal flank from anterior to posterior (Figure 1e). For these tag types and attachment sites tag-induced performance costs were estimated using both tagged and untagged mako shark geometries (2.95 m FL) at eight different flow velocities (Table 1).

### 2.7. Body Size Comparison

The effects of body size for a fixed tag size and tagging site were investigated. Here we examined MiniPAT impacts using tagging site 3 (Figure 1e) for mako sharks with four characteristic lengths of L = 1 m, 1.5 m, 2 m, and 2.5 m.

### 2.8. Impacts on Energy Expenditure and Swimming Speed

Daily energy expenditure for untagged sharks were estimated using the modelled drag forces applied to representative daily activity budgets, based on mako shark tracking literature. Mako sharks spend the majority of time cruising at speeds of 0.5–1 m/s [53,55], about 7% of their time in regular gliding (i.e., no active swimming) phases [55], and undertaking occasional burst-speed movements (3.6–5 m/s; [53,55]). Daily activity budgets of movement were therefore created by maintaining a constant 7% of time in gliding behaviour, varying the % of time allocated to burst speed movement from 0 to 20%, and assigning the remaining proportion of time to cruising behaviour (set at 1 m/s). Variations in time allocated to burst speed movement were used to account for variability across individuals and/or environmental contexts. Four scenarios were created with burst movement speeds of 3.6 m/s, 5.0 m/s, 7.8 m/s, and 9.1 m/s (Table 1) for the full-size mako shark model (i.e., 2.95 cm FL). Daily energy expenditure (E, in Joules) was then calculated as the sum of energy spent in cruising and burst-speed movement behaviour (energy expenditure during gliding was set to 0 as this behaviour does not involve active swimming), using Equation (8):

(8) E=time∗P

where *P* (power, in Watts) is:

(9) $$P=\;F_{drag}*speed$$

and *F_drag_* is the speed-specific drag force acting on the shark (in Newtons), estimated in simulations. A further four scenarios were created for the smaller model mako sharks, this time fixing burst speed at 3.6 m/s to account for the likely slower movements of smaller sharks. The modelling was then repeated for tagged sharks (both MiniPAT and SPOT tags separately for the full-size shark model, and MiniPAT only for smaller size sharks), comparing the relative increase in estimated daily energy expenditure.

Assuming that the power produced by the untagged shark at burst speed is the upper limit of the shark’s physical capability, and that swimming speed scales accordingly to accommodate for the increased energetic cost of drag added by the tag, tag-induced performance costs were estimated as the reduction in burst speed of a tagged shark necessary to produce the same power output of an untagged shark (according to Equation (9)).

## 3. Results and Discussion

### 3.1. Simulation Calibration and Testing

Simulations of mesh and modelling parameters against the DARPA SUBOFF reference provided support for the k-epsilon and k-omega SST turbulence models used in further simulations (Supplementary File S1B,C). The k-omega SST model predicted the net drag force more accurately than the k-epsilon model, generating small error values that fluctuated around the true value (delta force = −3.26 N–3.93 N vs. 5.55 N–85.80 N).

Simulations converged successfully within 3000 iterations for all cases with flow velocities above 1 m/s. For the lower flow velocities (≤1 m/s) convergence was less stable for the k-omega SST model, implying greater uncertainty in results from these simulations. However, results from the lower velocity cases appear to follow the general trends observed within the overall data, and the forces within this range are significantly lower than for the higher velocities, suggesting the absolute error may be less significant for this range.

### 3.2. Drag, Lift, and Pressure Force Distribution on Shark and Tag Geometries

**Figure 2:**
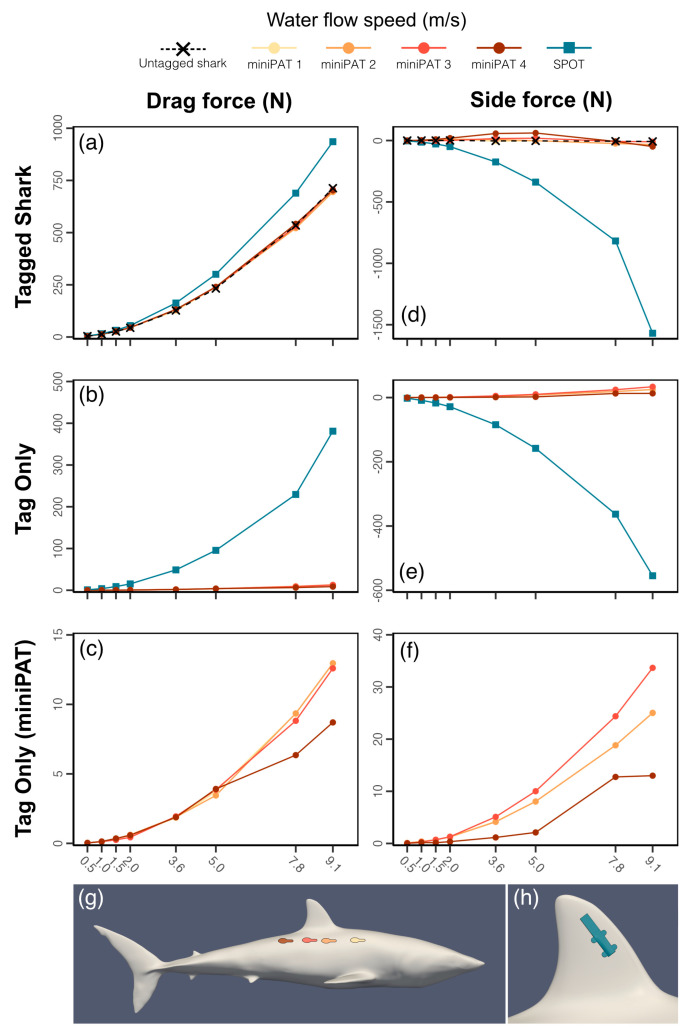
Tag effects on force balance. Drag (**a**–**c**) and side force (**d**–**f**) acting on tagged and untagged shark models (**a**,**d**) and on the tags in isolation (**b**,**e**,**c**,**f**) at sample swimming speeds. Bottom images (**g**,**h**) show tag placement of MiniPAT (**g**) and SPOT tag (**h**) and colours associated with lines.

Results from simulations of the shark geometry alone produced patterns in drag, lift, and side forces aligned with those of other modelling studies of sharks [59]. Drag and lift acting on the shark increased with water flow speed following a quadratic curve (Figure 2), with drag and lift coefficients varying little across the sizes tested (Table 2). Aerofoil surfaces were found around the pectoral and dorsal fins, and the pectoral fins were found to have a predominant role in generating lift.

### 3.3. Effect of Tag Shape and Attachment Position on Drag, Lift, and Pressure Forces

MiniPATs were found to have a negligible effect on the drag characteristics of the mako shark for all tagging sites tested across the full range of flow velocities used (Figure 2; Supplementary File S1D).

**Figure 3:**
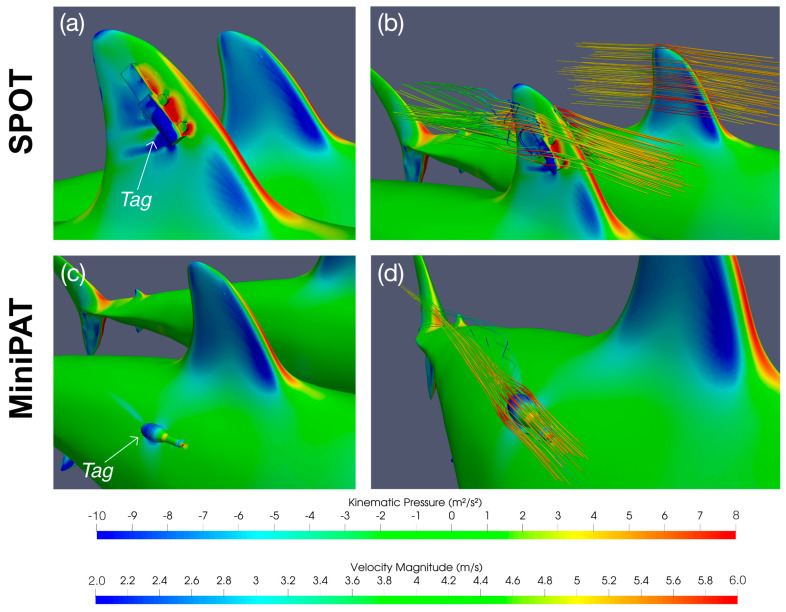
Tag effects on water flow. Kinematic pressure (**a**,**c**) and water flow velocity ((**b**,**d**); represented by sample particle movement lines) around SPOT tags (**a**,**b**) and MiniPATs (**c**,**d**). Colours on the shark and tags bodies indicate pressure, colours on sample flow lines indicate water velocity.

The largest overall increase in drag force was found to be 7.9 N for tagging position 4 (furthest location behind the first dorsal fin) at flow speed 0.5 m/s, which is small compared to the drag of the untagged shark (3.4% increase). MiniPATs are therefore likely to have a minimal effect on the resistance of a shark to the water flow and hence impose minimal energetic cost for tagged animals. The limited impact of this tag can likely be explained by its streamlined design that is aligned with the water flow direction, and which does not protrude beyond the shark’s maximum cross-sectional area when deployed at the tagging positions considered here (Figure 3).

In contrast, the dorsal fin SPOT tag was found to increase the drag experienced by the shark, with a 0.7–222.7 N (17.6%–31.2%) increase in drag force across the range of flow velocities, increasing for increasing flow velocity (Figure 2; Supplementary File S1D). MiniPAT tags remain within the upstream flow boundary of the shark’s body, minimizing exposure to direct flow and reducing drag. The SPOT tag is instead attached to the dorsal fin, where it disrupts the aerofoil-like geometry and protrudes beyond the original cross-sectional area of the shark, and as such is not shielded by upstream flow structures (Figure 3).

As the addition of the tag changes the water flow around the shark body (Figure 3), alterations in forces other than drag may also impact swimming performance. The change in the side force represents the most significant change to the overall force balance on the shark (Supplementary File S1D). The dorsal fin SPOT tag, in particular, creates an increase in the absolute force acting against the side of the dorsal fin (Figure 3), estimated to be as high as 106 N at flow speed of 9.1 m/s (the area is subject to side forces of −9.7 N in the untagged case). The flow characteristics around the dorsal fin on the side of the tag are also disrupted (Figure 3), while the flow around the untagged side remains largely unchanged. The imbalance allows for a higher velocity flow around the untagged side and leads to a lower pressure area on this side. As a result, the pressure difference between the two sides of the fin causes the overall force to act in the opposite direction, tending to cause changes to the control/stability characteristics of the fin. As the dorsal fin functions primarily as a stability control device [60,61,62], it is possible that the pressure imbalance may introduce some level of disruption to the overall stability and swimming performance of the animal.

The impact of the MiniPAT on side force distribution and intensity was more variable, with differences in force magnitude between tagging position scenarios, although always much smaller than that observed for SPOT tags (maximum force 64 N vs. 106 N). Tagging position 4 produced the greatest absolute change in side force to the shark (a maximum of 64 N), and tagging position 3 produces the smallest absolute change across the range of flow velocities (a maximum of 22 N; Figure 2). However, the side force acting on the tag surface (exerting force that may dislodge the tag from the body contour) was lowest for tagging position 4, and greatest for tagging position 3 (13 vs. 34 N). Similarly, the two placements have somewhat different impacts on the wake characteristics of water flow: the wake from tagging position 3 appeared to interact more strongly with the second dorsal fin, which could alter stability characteristics, but also appeared to produce a wake that symmetrically enveloped the tail, potentially stabilizing thrust. The wake from placement location 4 instead followed the side of the body and flowed past one side of the tail, inducing lateral wake asymmetry which could create an imbalance in thrust generation. It is difficult to accurately predict the interplay of such forces from stationary simulations; nonetheless, the absolute magnitude of the forces estimated suggest that differences between placements are likely minimal and may not result in biologically appreciable changes. Further analysis for a wider range of conditions, such as modelling the caudal fin thrust generation or testing for a range of pitch and yaw angles may provide more insight as to the optimal choice between these two placements.

Lift characteristics remained largely unchanged for any of the tagged cases, as neither of the tags are situated on or near the pectoral fins, which largely dominate lift force generation in the animal.

### 3.4. Considering Animal Size

While accurately selecting shape and placement of electronic tags can help to minimise the impact on study animals, researchers should also consider the relative size of the tag package compared to the intended study species. Often, technology development has primarily focused on tag miniaturisation, as this is likely to allow for a wider range of applications. However, the scaling relationship of energetic cost and animal size remains poorly understood, and it is hence challenging to determine what animal sizes tags are currently fit for.

**Figure 4:**
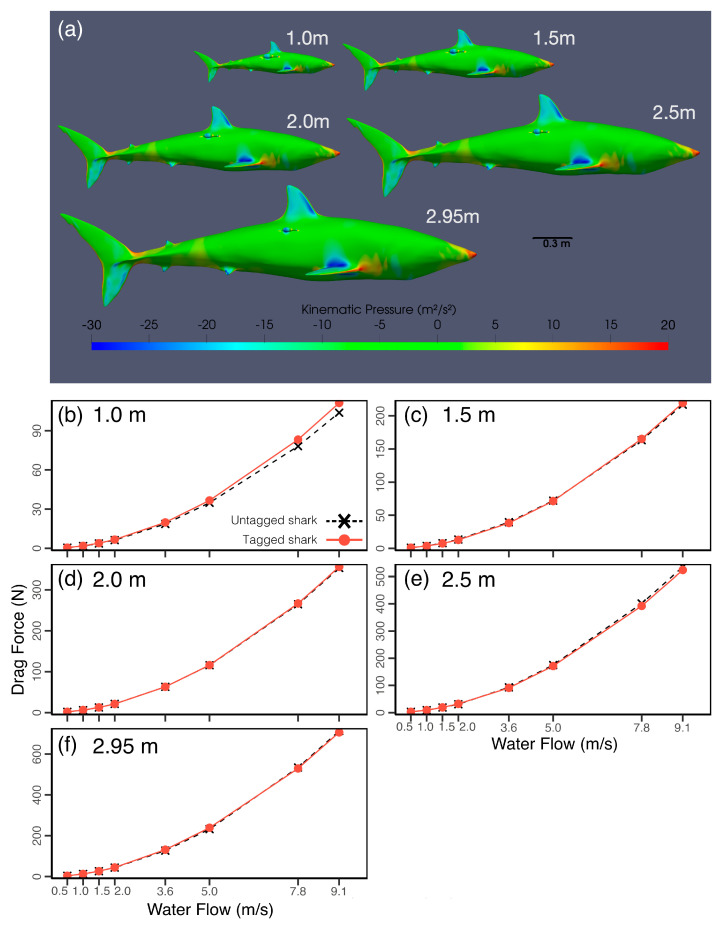
Forces acting on MiniPATs on sharks of different sizes. (**a**) Kinematic pressure resulting from water flow around mako sharks of varying sizes tagged with MiniPATs. (**b**–**f**) Drag force acting on tagged (continuous line) and untagged (dashed lines) mako sharks of various sizes. Note varying *y*-axis limits.

In simulations, the overall trends in drag force across the range of flow velocities tested followed a similar relationship for a range of shark sizes from 1 to 2.95 m (Figure 4). When MiniPATs were at position 3, a significant increase in drag was identified for the smallest size of L = 1 m (Figure 4). For this size, the absolute difference in drag between the tagged and untagged cases positively scales with flow velocity, with a maximum observed increase of 7. 5 N for flows of 9.1 m/s, a 7.2% increase in relation to the untagged drag force (Figure 4). This change remains moderately constant throughout the range of velocities tested, varying from 5.1% at 5.0 m/s to 7.6% at 0.5 m/s. In comparison, the maximum increase in drag observed for the next larger test size (L = 1.5 m) was 2.6 N, or 1.2% of the untagged case.

For the range of shark sizes and flow velocities tested, the net force acting on the tag fluctuated around similar values independently of shark size. Even in the smallest case, the MiniPAT did not increase the maximum cross-sectional area of the shark, suggesting that, for this tag size and placement, the drag force on the tag is likely to remain relatively constant, so long as the tag does not protrude beyond the shark’s maximum cross-sectional area. Although absolute drag values remain consistent, their proportional impact increases in smaller sharks, potentially amplifying energetic or stability costs.

The pitching moment coefficient was analysed for the range of shark body lengths tested, and it was found that although the tendency to rotate was small, the change in moment between the untagged and tagged cases became more pronounced for L = 1 m and L = 1.5 m, showing that for a smaller shark the tag may induce a change in the shark’s angle of attack, which would further change the motion and drag characteristics of the tag and may also affect thrust generation and maneuverability. Other forces showed a greater complexity of interaction between the fixed tag and varying shark size across the range of flow velocities tested (Supplementary File S1E) and would likely increasingly impact the shark for smaller sizes. Though these changes may not directly increase energy expenditure, they show the potential to disrupt the stability and direction of locomotion.

The simulations presented did not address possible changes in body shape with ontogeny, instead assuming that small sharks retain the same proportions as adults. While small morphological variations can change resulting flows, key flow features are expected to remain persistent. As such, the differences in flow disruption and energetics discussed here are likely to persist in small ontogenetic changes in body shape.

Small and/or young sharks display higher metabolic rates and lower energy reserves than adults [63], making them particularly susceptible to additional energetic losses. The energetic costs of external tags estimated in this study may lead to decreased hunting success rates for juvenile sharks, in turn retarding growth and possibly reducing survival rates.

### 3.5. Energetic Consequences of Tagging

**Figure 5:**
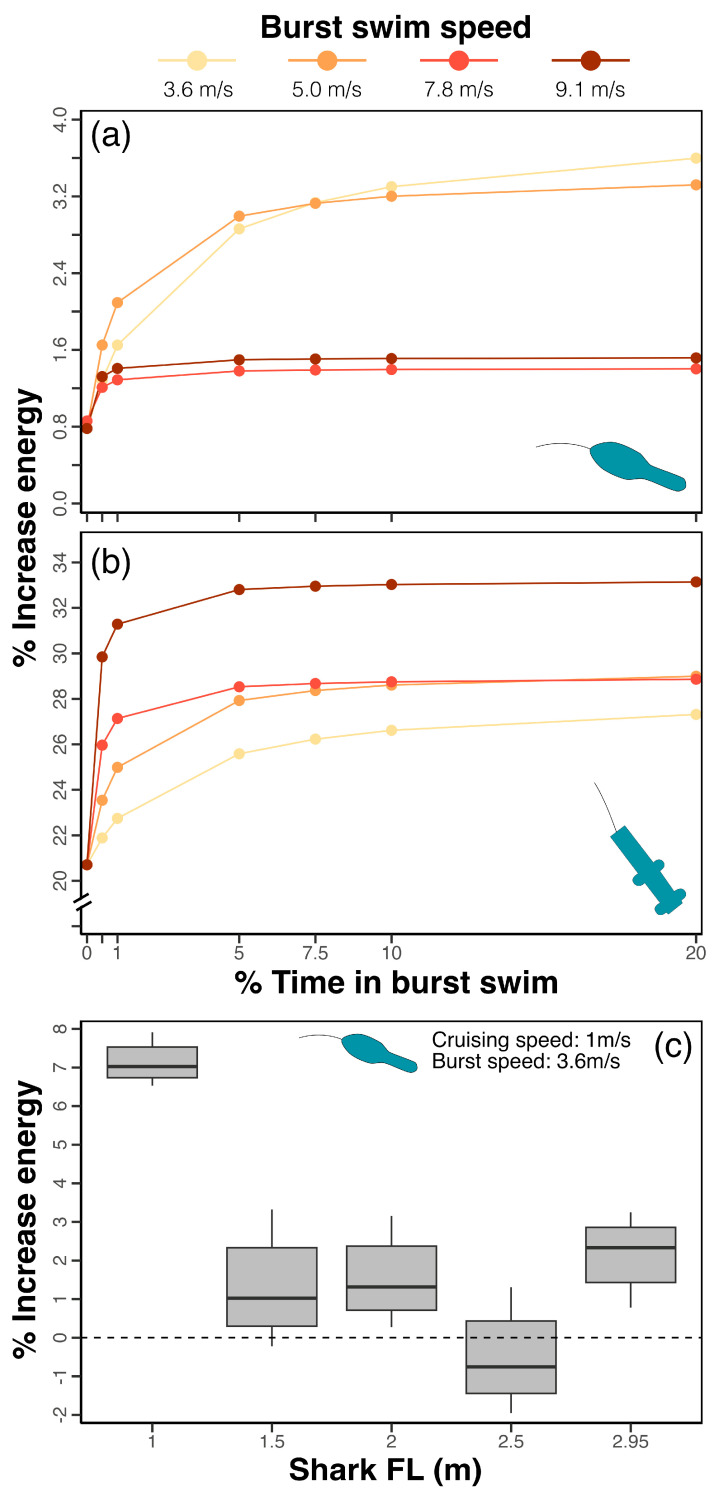
Energetic cost of tags. (**a**,**b**) Percent increase in daily energetic cost of movement for a modelled 2.95 m adult mako shark (FL) tagged with a MiniPAT (**a**), or fin-mounted SPOT tag (**b**), with increasing time spent burst swimming at various measured speeds (indicated by colour). (**c**) Quantiles and median percent increase in daily energetic cost of movement for modelled mako sharks of varying size with a fixed burst speed of 3.6 m/s, cruising speed of 1 m/s, and varying % of time in burst movement.

Drag force alone does not fully characterize the cost of carrying electronic tags, as energy expenditure (and the relative change in it owing to drag) also depends on swimming behaviour (i.e., speed, which also affects drag magnitude) and time (the duration of the behaviour of interest). To estimate the additional energetic cost of carrying electronic tags, we simulated various daily activity scenarios that included cruising behaviour (i.e., swimming at constant, low speed of 1 m/s), gliding (unpowered, gradual descending movement), and burst swim events, in varying time allocations. For the largest mako shark model (2.95 m), both tag types led to an average 1.02 and 1.27 times increase (MiniPAT and SPOT tag, respectively) in estimated energetic expenditure under all combinations of the three behaviours (Figure 5a,b); however, MiniPATs led to an energetic cost an order of magnitude smaller than that caused by SPOT tags (232 ± 352 kJ vs. 4184 ± 7483 kJ). Daily estimated energetic cost was smaller when less time was dedicated to burst swimming, but increased rapidly with time spent in this behaviour, plateauing when burst swimming constituted 5% or more of the daily activity budget (Figure 5a,b).

MiniPAT additional energetic costs ranged from 0.8% to a maximum of 3.5% for the lowest burst swim speed scenario (i.e., 3.6 m/s). Above this burst speed, the maximum energetic cost of MiniPATs was lower (1.4% and 1.5% for 7.8 m/s and 9.1 m/s scenarios, respectively; Figure 5a,b), as the drag force acting on the tag did not increase with water flow by as much as the relative drag caused by the shark itself. By contrast, SPOT tag costs varied from 20.5% (in the absence of burst swimming), up to 33% for the 9.1 m/s burst speed scenario. Energetic costs associated with SPOT tags furthermore increased with increasing burst speed (Figure 5a,b).

The energetic cost of MiniPATs in the present study was similar across shark sizes (Figure 5c), with the exception of the smallest case (i.e., 1 m FL). For very small sharks, it appears that the smaller, more streamlined MiniPAT can produce an energetic demand increase of 7.1 ± 0.5% (mean ± standard deviation), which may not be trivial to an already slow-growing animal [64]. Only two other studies have attempted to calculate the energetic cost of carrying electronic tags for sharks [35,65]. While the knowledge base is small, the results of those studies broadly agree with the predictions of the present modelling exercise, with MiniPAT-like tags having no appreciable impact on the metabolic rate of juvenile sandbar shark (*Carcharhinus plumbeus* [35]), while fin-mount tags led to a 4–25% increase in oxygen consumption rates for blacktip sharks (*Carcharhinus limbatus* [65]).

Dolphins and seals reduce their maximum swimming speed in response to tag drag [39]. Assuming that recorded mako shark burst speeds represent the maximum power achievable by the shark, the addition of tag drag would also cause these animals to reduce their speed. The simulations presented in the present study suggest that burst speed movements for an adult (2.95 m FL) mako sharks would be reduced by 1.4–3.7% (0.1–0.2 m/s slower) by the attachment of a MiniPAT, and by 22.4–22.7% (1.1–2.3 m/s) due to SPOT tag attachment. Mako sharks feed on fast-moving animals [61,66,67,68] and are thought to rely on speed to maximize predation success, although their diet is varied and includes slower-moving prey [66,69,70]. A reduction in burst speed may impair the animal’s hunting, compounding the impacts of increased cost of movement.

### 3.6. Limitations of Modelling

The simulations presented in the study are based on some fundamental assumptions on the nature and behaviour of shark geometry and water flow, namely that the shark can be represented by a rigid body with a constant flow velocity, the existence of a fully turbulent boundary layer, and that the dermal denticle structure has no influence on water flow (i.e., the shark is assumed to be a smooth surface). These simulations do not incorporate body undulation, caudal fin propulsion, or behavioral compensation, which may modulate the observed force distributions. As such, the results presented here may only approximate real-world conditions and may underestimate the effects of tags on swimming performance. In studies where CFD simulations were compared directly to in-vivo measurements of drag or energetic expenditure of tagged animals, model predictions were found to closely match real-world scenarios, though simulated drag increases sometimes underestimated the real effect of tags [7,39] and suggest that static approximations of movement from linear water flow do not fully represent real world scenarios. For example, the wake characteristics of the tags modelled in this study showed some interaction with the caudal fin flow for many of the cases, which may result in changes in thrust generation once tail movement is considered. Further, the microstructure created by dermal denticles is known to affect the hydrodynamic balance of sharks [59,71], though the effect of these structures could not be included in present models and may exacerbate the impact of the added drag from the tags. With the development of CFD techniques and improvement of computing power, simulations that can account for these and other parameters should become more readily available and provide further insights into optimal tagging methods. Future work should incorporate dynamic swimming simulations, caudal fin thrust modeling, and in vivo validation to assess behavioral compensation and tag retention. Nonetheless, given the simulations presented here closely match the calibration case and provide similar flow characteristics to those simulated for this species [59,71], they may already offer useful insights into the practice.

### 3.7. Relevance to Tagging Practice

CFD simulations suggest that both the choice of tag and tag placement can impact on the hydrodynamic force balance of a tagged animal. The observed force imbalances caused by tag attachment may lead to compensatory muscular effort or altered swimming kinematics in sharks, potentially influencing energy expenditure or migratory efficiency. Overall, the fin-mounted SPOT tag was estimated to have a greater impact on the hydrodynamic force balance of the shark than dorsally-attached MiniPATs, causing an increase in drag force of 17.6–31.2% and significant energetic costs for the animals. The rigidity and protrusion of SPOT tags likely exacerbate flow disruption, whereas the streamlined MiniPAT design mitigates such effects. The SPOT tag was also found to strongly alter the pressure characteristics over the side of the dorsal fin to which it is attached, creating a large (up to 1670 N) side force on the shark. The drag and side forces acting on the tag may tend to cause an increased chance of premature tag shedding and possible damage to the fin due to the high loading on the attachment points for the tag (e.g., [19,72]). This type of tagging may therefore not be optimal for fast swimming species, for which these forces become greatly magnified. While these tags are often crucial in obtaining high-quality location data for marine taxa, the potential impacts of their current shape and placement on hydrodynamic balance requires careful ethical consideration. This is especially true for “DIY” tag packages that are often fin-mounted, of larger dimensions than the SPOT tag examined here, and have been through fewer stages of refinement in shape (in comparison to established tags with greater use).

For the MiniPAT, the range of placement locations tested were not found to lead to a tangible increase in the drag experienced by large (2.95 m FL) sharks (max. drag increase = 3.3%), and its overall impacts to the force balance were overall relatively small, though this is dependent on flow velocity and tag placement. Placement location 3 and placement location 4 show some advantages over placement location 1 and 2, such as the reduced drag on the tag surface for higher flow velocities with placement location 4, and the lesser impact to the side force acting on the shark throughout the range of flow velocities for placement location 3. This suggests that, although the range of MiniPAT placements are reasonable choices for tagging, centring the tag near the back of the dorsal fin or slightly behind it may be the optimal choice. This placement produces minimal or undetectable changes to the overall drag of sharks larger than 1.5 m.

For sharks smaller than 1 m FL, however, a significant increase of 5.1% to 7.6% in drag force due to tagging was observed, leading to an increased cost of transport for the animals, which may detract from somatic growth. A better understanding of the appropriate tag characteristics for small animals is needed in the marine realm, but the results of the present study suggest that a lower size limit for animals entering tagging procedures should be considered in any telemetry study.

Tag to animal weight ratios have often been used to determine thresholds for tagging, with the clearest example found in the 3% rule first proposed in avian studies [20]. These relationships have also been used in marine systems [21,22,23], and have sometimes been criticised due to the difficulty in translating weight in air or in water to a metric relevant to underwater locomotion. In the context of this study, for example, the estimated weight of a 1 m FL mako shark can be estimated at 17 kg [73]; as such, a MiniPAT (weight in air of 60 g) would only equate to 0.35% of the shark’s body weight and hence be acceptable under the 2% rule of thumb sometimes adopted in the field. However, the simulations presented here appear to contradict this and show that the energetic costs associated with the tag are disproportional to its mass. Similarly, the relatively small SPOT tag may produce enough drag to considerably increase the energetic cost of movement of an adult-size shark. As the difference in energetic cost between tagged and untagged animals were found to be greater at lower movement speeds, such impacts could be felt even by slow-moving species, not just burst predators.

Both tags have been used on pelagic sharks of varied sizes, with apparent success in data collection [32]. However, it is impossible to know whether the data collected by these tags represents “natural” behaviour or an attempt by the shark to compensate for the impact of the tag. Determining the long-term effects of tags on the animals is equally challenging. Mako sharks and other lamnid sharks are thought to have a higher metabolic scope than most other sharks and teleost fish [74], and may hence be able to cope with some increase in cost of movement. However, the hunting strategies of the species relies on speed to maximise success, so that the reduce burst speed resulting from the increased drag afforded by the tag may reduce predation success and negate the energetic advantage of a higher metabolic scope.

Other pelagic species, including the blue shark (*Prionace glauca*) or the oceanic whitetip shark (*C. longimanus*), instead rely on energetic strategies that seek to minimise the cost of transport to maximise the energy gained form prey [75]. For these animals, energetic costs like the ones predicted in the present study may have consequences for fitness.

## 4. Conclusions

The guiding principles of the 3Rs (Reduce, Reuse, Refine; [76]) require scientists to critically think about how the field of biologging may be continuously improved to ensure the highest quality of scientific products while maximising welfare of animals. Refinement of tagging procedures should be, therefore, of particular importance to the wider community. Understanding and mitigating the potential negative impacts of tag load is not only an ethical and moral duty towards the animals employed in biologging studies, but also an important factor in determining data quality. The CFD-based simulations presented in this study demonstrate that tag-induced hydrodynamic costs vary by tag type, placement, and body size, underscoring the need for biologically informed deployment strategies.

We recommend that tag manufacturers and the biologging community collaborate to develop easily accessible and interpretable tools that researchers can use to estimate and evaluate more holistically the harm:benefit arising from studies before they commence. Such tools must be grounded in empirical data derived from both CFD modeling and in vivo performance metrics to ensure ecological relevance. For example, reference tables describing estimated energetic effects of typical tag shapes at various swimming speeds could be created for a variety of shark body forms, and used as a primary tool in assessing feasibility of tagging studies (e.g., see Supplementary File S2). Ethical thresholds for the field are yet to be determined and will require a better understanding of sharks’ energetic budgets and physiology. Thresholds may include maximum allowable drag increase, side force asymmetry, or pitching moment deviation, calibrated against species-specific energetic budgets. Nonetheless, as shark tracking studies rapidly increase in number [32] and evidence for the hydrodynamic impacts of tags on marine animals accumulates (e.g., [28,29,65,77]), it is clear that more coherent ethical standards are needed, based on metrics that are relevant to this realm and founded in empirical data.

## Figures and Tables

**Table 1:** Mako shark swimming speeds used for modelling, given fork length (L) of 2.95 m.

Inlet Velocity $U_{\infty }$ [m/s]	Reynolds Number	Source
0.5	$1.39\times 10^{6}$	[52,53,54]
1.0	$2.77\times 10^{6}$	[54,55]
1.5	$4.16\times 10^{6}$	[52]
2.0	$5.55\times 10^{6}$	[54]
3.6	$9.99\times 10^{6}$	[53]
5.0	$1.39\times 10^{7}$	[55]
7.8	$2.16\;{\times \;10}^{7}$	[54]
9.1	$2.52\times 10^{7}$	[54]

**Table 2:** Drag coefficient (*C_D_*) of tagged and untagged (“No tag”) sharks of varying size (Fork Length) obtained from simulations.

	Speed (m/s)	C_D_ 1.0 m	C_D_ 1.5 m	C_D_ 2.0 m	C_D_ 2.5 m	C_D_ 2.95 m
No Tag	0.5	0.138	0.128	0.123	0.123	0.121
	1	0.118	0.110	0.100	0.097	0.098
	1.5	0.110	0.104	0.093	0.089	0.089
	2	0.105	0.099	0.088	0.082	0.084
	3.6	0.096	0.090	0.081	0.076	0.074
	5	0.093	0.085	0.077	0.074	0.071
	7.8	0.085	0.079	0.072	0.070	0.067
	9.1	0.083	0.077	0.071	0.069	0.066
MiniPAT	0.5	0.148	0.131	0.126	0.124	0.123
	1	0.120	0.107	0.103	0.099	0.098
	1.5	0.117	0.098	0.093	0.090	0.090
	2	0.111	0.094	0.088	0.086	0.085
	3.6	0.102	0.087	0.081	0.075	0.078
	5	0.097	0.084	0.077	0.073	0.073
	7.8	0.091	0.080	0.073	0.069	0.066
	9.1	0.089	0.078	0.071	0.067	0.065
SPOT	0.5	-	-	-	-	0.143
	1	-	-	-	-	0.118
	1.5	-	-	-	-	0.109
	2	-	-	-	-	0.104
	3.6	-	-	-	-	0.096
	5	-	-	-	-	0.092
	7.8	-	-	-	-	0.086
	9.1	-	-	-	-	0.084

## Data Availability

The raw data supporting the conclusions of this article will be made available by the authors on request.

## Supplementary Materials

The following supporting information can be downloaded at: https://www.mdpi.com/article/10.3390/ani15202956/s1, Supplementary File S1: additional details on modelling processes and results; Supplementary File S2: example lookup table for ethical appraisal of shark tagging studies; Supplementary File S3: simulation outputs for the untegged case; Supplmentary File S4: simulation outputs for case 1 (effect of tga shape and placement on hydrodynamic forces of a mako shark); Supplementary File S5: simulation outputs for case 2 (effects of miniPAT tags on mako sharks of different sizes

## Abbreviations

The following abbreviations are used in this manuscript:

CFD	Computational Fluid Dynamics
FL	Fork Length
