# Supplementary material for: Refining Electronic Tagging of Marine Animals: Computational Fluid Dynamics and Pelagic Sharks
Source: Animals (Basel). 2025 Oct 13;15(20):2956. doi: 10.3390/ani15202956 (PMC12560902; doi:10.3390/ani15202956)
Supplement: Supplementary file 1 [file animals-15-02956-s001.zip › FileS1_MethodologicalDetails.pdf]

Refining electronic tagging of marine animals: computational fluid dynamics and pelagic sharks

Tobias Maillard, Francesco Garzon, Lucy A. Hawkes, Gavin Tabor and Matthew J. Witt

Supplementary Material S1

Supplementary A. Mesh generation with snappyHexMesh

A.1 Background Mesh Generation with blockMesh

The first step of the mesh generation process was to create a background mesh of hexahedral cells using blockMesh, a utility provided by OpenFOAM that can create simple meshes from a dictionary file (Greenfields, 2022a). The domain had dimensions of (16 x 8 x 8) m, with the shark's centre at the origin, using 8 blocks split symmetrically about the origin, each with a grading factor applied to allow for an increasing mesh refinement towards the origin in all directions. Inlet and outlet boundary patches were created, as well as identifiers for the unused domain walls. As part of the refinement process, the base mesh density was increased for each refinement level to create a finer background mesh.

A.2 Mesh Refinement with snappyHexMesh

The second step of the mesh generation process was to castellate and snap the background mesh to the shark geometry using snappyHexMesh, an OpenFOAM utility that can create high quality hexahedral-dominant unstructured meshes from triangulated surface geometries (Greenfields, 2022b). SHM consists of three main steps: castellation, snapping, and layer addition. This study only used the first two steps, as layer addition was not necessary for the wall model-based approach given the sufficient surface refinement. Moreover, layer addition often resulted in errors that degraded the mesh quality.

The castellation step creates a mesh of hexahedral cells that fit the geometry by splitting and removing cells that intersect with the surface. The snapping step moves the vertices of the castellated mesh towards the geometry surface to improve the alignment and accuracy of the mesh, and also modifies the cell connectivity and topology to avoid overlapping or distorted cells. The complexity and curvature of the shark geometry required careful tuning of the SHM settings to ensure a successful meshing; it is estimated that at least 75% of all mesh configurations failed.

The meshing process with SHM used a pseudo 'genetic algorithm' approach. This approach involved determining mesh settings based on initial research (Greenfields, 2023; Jackson, 2011; Wolf Dynamics, n.d), then running batches of meshing, and selecting only the most successful original meshes to be 'parent' meshes, where pseudo random mutations were applied to the mesh settings (based on the knowledge of what each setting should do) to iterate and improve mesh quality until acceptable meshing was achieved. The details of how the mesh settings were modified for each refinement level are explained in the following subsections.

Some key settings that were found to be important for achieving an accurate representation of the surface geometry are listed below:

- Max Global cells and Local cells (cells per processor) must be sufficiently high to not limit the meshing process.

- "minRefinementCells" was kept low to allow refinement of all cells, as often a few cells were causing errors which cause the entire mesh to fail.
- "nCellsBetweenLevels" was kept at around 3, to allow smooth growth between different refinements; too low and the aspect ratio between adjacent cells became too large.
- Explicit feature edge refinement was useful in achieving a higher degree of geometric accuracy for the finer meshes. It snaps the mesh to the feature edges, which are edges with an angle less than the 'includedAngle' between the adjacent surface normal (optimum was found at around 170 degrees). This captures the sharp angles and curves in the geometry, improving the accuracy and quality of the CFD simulation by reducing the mesh distortion and alignment close to the surface, and was implemented for the finest meshes using a level 6 refinement.
- Surface based refinement was a key parameter used to achieve an accurate representation of the surface geometry; this was set to (5 x 6) for the finer cases and (4 x 5) for the coarser cases. The 'resolveFeatureAngle' specifies which cells intersecting the surface of the shark are treated with the higher level of refinement; using a low angle is crucial to capturing the high curvature gradients of the geometry, especially near the 'sharp' trailing edges of the fins, which were a considerable source of erroneous cells, as shown in Figure S1. An angle of 5-10 degrees was found to be optimal for capturing the curvature of the shark, with higher values reducing the accuracy of the mesh in the highly curved regions.
- A refinement box (between levels 1-2) was used for all mesh settings; as the main area of interest was around the shark, allowing for a coarser background mesh to be used; the box was centred around the shark, but extended further behind the shark to account for the wake produced by the shark.

As finer meshes were implemented, the number of iterations for each stage was increased; this includes more patch smoothing, mesh displacement relaxation, and snapping relaxation iterations, which improve the mesh quality and reduce the distortion and non-orthogonality. The fine meshes were also set to a lower relative tolerance value, which results in a more accurate snapping of the mesh to the geometry.

For the finer meshes, the strictness criteria of the "meshQualityControls" (skewness, tet quality, twist, determinant, face weight, and volume ratio) were increased, while the minimum volume was scaled appropriately to account for the smaller cell sizes.
This resulted in less distorted, more orthogonal, more uniform, and more balanced cells, which improved the CFD solution quality and convergence.

The mesh refinement process is illustrated in Figures S1–S3

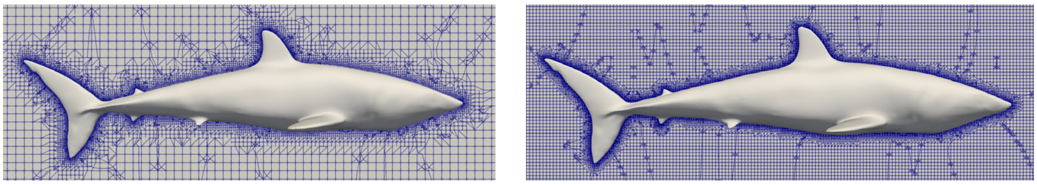

Figure S1. Mesh refinement – cell size comparison. Showing (left) Mesh 2, and (right) Mesh 5, highlighting the overall difference in refinement levels.

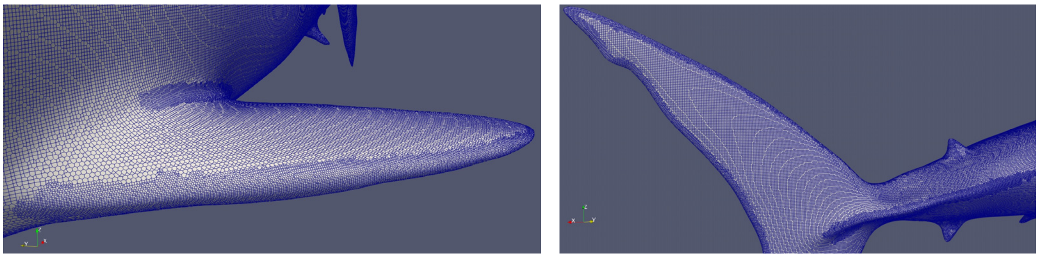

Figure S2. Mesh refinement – high curvature regions. Showing Mesh 5, highlighting the benefit of a low 'resolveFeatureAngle' setting to improve the quality of the surface mesh by capturing regions of high curvature through increased refinement, such as at the edges of the fins.

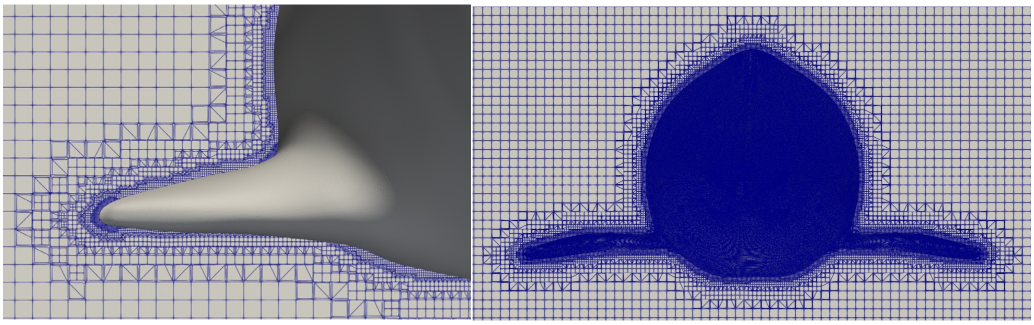

Figure S3. Mesh refinement – Boundary layer. Mesh 6, showing even boundary layer meshing, achieved through a suitable surface refinement level (5 6) and use of a reasonably high "nCellsBetweenLevels" to allow smooth growth between adjacent cells.

A.3 Mesh Quality Inspection and Automation

The final step of the mesh generation process was to inspect and evaluate the quality of the mesh using "checkMesh" command. This command checks various aspects of cell quality such as aspect ratio, skewness, non-orthogonality etc. Erroneous mesh properties were inspected visually in ParaView by running the command "runParallel checkMesh -allGeometry -allTopology -writeSets vtk" so that features that caused errors could be identified, inspected and improved, as shown in Figure S4. Final meshes were selected based on key mesh indicators, such as being visually pleasing, having cell faces aligned with expected flow direction, ensuring that the shark surface closely matched the mesh with nice even boundary layer meshing, and having most of the cell refinement focused around the shark. The full meshing process was automated and run in batches using a custom bash script to loop through each case folder and complete this process.

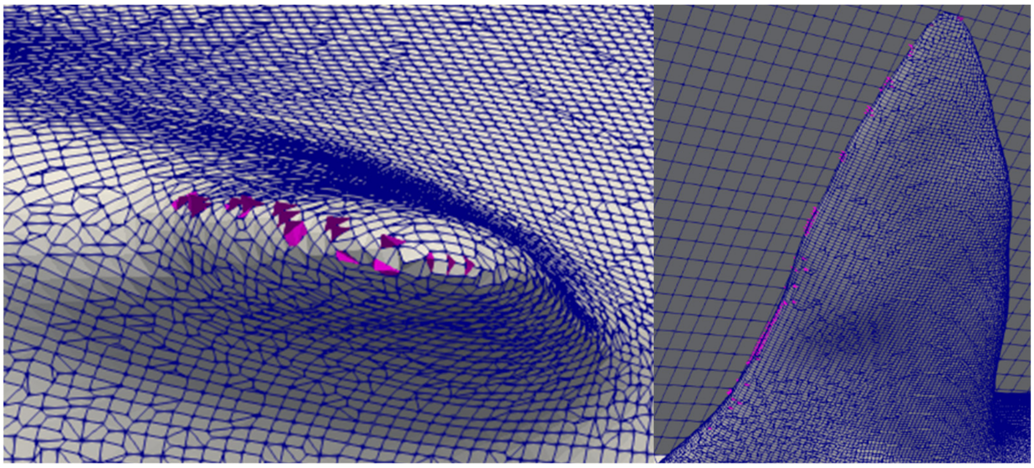

Figure S4. Cell warping and simulation failure. A visualization of warped cell faces which resulted in simulation failure. *S*howing that much of the mesh failure was due to the strong curvature of the geometry around the fins (especially at the 'sharp' trailing edges).

Supplementary B. Mesh independence study

The mesh independence study aimed to determine the optimal mesh resolution for the CFD simulations using the simpleFoam solver with the k-ε and k-ω SST turbulence models. This involved measuring the key parameters of interest, namely the net viscous and net pressure forces acting on the shark in the x, y, and z directions, to identify where no changes occurred for an increasingly fine mesh, indicating that the solution was independent of the spatial discretization error. The numerical residuals were also monitored, especially the pressure residual, which was the most difficult to converge. The choice of conversion tolerance was decided using a graph of the logarithm of the residual values against the iteration number; the convergence tolerance value was selected as the point where the initial pressure residual levelled off and did not decrease for further iterations.

The net forces in each direction were recorded from the final iteration of each simulation, and were compared by plotting these values against the number of cells. The plots were assessed visually and the absolute and percentage changes between adjacent values were also calculated; the aim was to identify the least costly mesh that produced a mesh-independent solution, so that it could be used as the most efficient mesh for further study.

The simulations used the original shark geometry (L = 2.95 m) with free stream velocity, $U_\infty = 9.1\ m/s$, and a turbulence intensity of 2%. The boundary conditions given in Table S1 were applied for each case, using the automatically calculated spreadsheet values. The k-ε runs were initialized using the potentialFoam potential flow solver (OpenFOAM, n.d.), and the k-ω SST runs were initialized using the converged solution of the k-ε simulations for the same mesh.

Based on initial simulations, the convergence tolerance was set to an initial pressure residual value of 5.5×10^{-4}, which is corroborated within Figure S5, which shows representative residual plots for the convergence of the simulations. The mesh independence analysis was conducted by comparing the forces for each mesh resolution and selecting the optimal mesh that showed the least relative change between adjacent values, and by observing the overall stability of the forces. The relative changes are summarised in Tables S2 and S3.

The results show that the pressure force in the lift direction is the most sensitive to the mesh resolution and the turbulence model. As seen in Figures S8 and S11, this force exhibits some oscillatory behaviour which does converge strongly within the range tested. A possible reason for this behaviour is the influence of the large pectoral fins (wings) that have an angle of attack when the main body of the shark is aligned with the flow direction. This may introduce some time-dependent dynamics that are not captured by the steady-state solver or imply some numerical error within the domain. However, the good convergence of the other forces suggests that this is due to physical instabilities rather than numerical issues.

The viscous forces in all directions show strong convergence for both the k-epsilon and k-omega SST turbulence models, as shown by the relative changes between meshes 3-7 in Table S2 and meshes 5-7 in Table S3 for the k-epsilon and k-omega SST models, respectively (highlighted for the x-direction in Figures S6 and S9). The viscous and pressure drag forces also exhibit strong convergence for both cases, as shown in Figures S6, S7, S9, S10. Considering that this was the main parameter of interest for this study, this was the key indicator used to select the mesh, with the other forces considered as secondary parameters.

Based on these results, Mesh 5 was selected to be used for the k-epsilon model, and Mesh 6 was chosen for the k-omega SST model, as they showed satisfactory convergence and accuracy for all forces and produced the least overall relative change between adjacent mesh refinements.

Table S1. Applied boundary conditions for the computational domain

Quantity	Boundary	Type	Value
Uniform free-stream velocity, $\boldsymbol{U}_\infty$	inlet	fixedValue	uniform (U_∞ 0 0)
	outlet	zeroGradient	
	defaultFaces	symmetry (slip condition)	
	MakoShark	fixedValue (no-slip)	uniform (0 0 0)
Kinematic pressure, p	inlet	zeroGradient	
	outlet	fixedValue	uniform 0.0
	defaultFaces	symmetry (slip)	
	MakoShark	zeroGradient	
Turbulent kinetic energy, k	inlet	fixedValue	uniform k
	outlet	zeroGradient	
	defaultFaces	symmetry (slip)	
	MakoShark	kqRWallFunction	uniform k
Turbulent dissipation, ε	inlet	fixedValue	uniform ε
	outlet	zeroGradient	
	defaultFaces	symmetry (slip)	
	MakoShark	epsilonWallFunction	uniform ε
Turbulent viscosity, μ_t	inlet	calculated	uniform 0
	outlet	calculated	uniform 0
	defaultFaces	symmetry (slip)	
	MakoShark	nutkWallFunction	uniform 0
Turbulent frequency, ω	inlet	fixedValue	uniform ω
	outlet	zeroGradient	
	defaultFaces	symmetry (slip)	
	MakoShark	omegaWallFunction	uniform ω

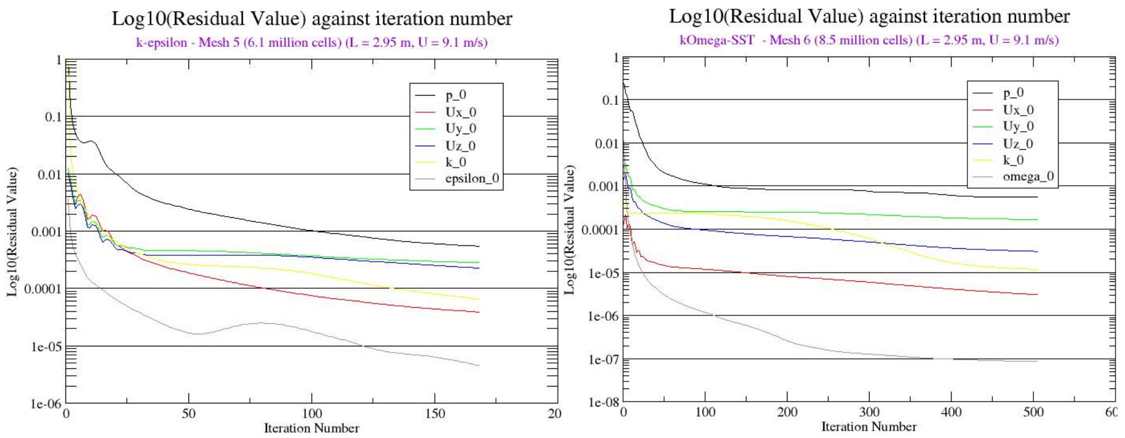

Figure S5. Residual plots for the convergence of the simulations. Showing residual plots for a representative k-epsilon (left) and k-omega SST (right) simulation; the pressure residual has the highest convergence tolerance, and levels off at a value of around 5.5E-04, which is selected as the main convergence criteria for further simulations.

B.1 k-epsilon results

Table S2. Results from the k-epsilon simulations for mesh 1-7.

Mesh	No. of cells	Average Y+ at walls	Direction	Sum of pressure forces [x y z] (N)	Sum of viscous forces [x y z] (N)	Relative change from previous value: pressure (1) [z y z], viscous (2) [x y z]	
1	261,407	390.8	x (drag)	807.7	339.2	~	~
			y (yaw)	-123.7	0.1	~	~
			z (lift)	1288.77	-7.1	~	~
2	1,010,406	248.9	x	585.6	410.7	-222.1	71.5
			y	87.5	-0.1	211.2	-0.2
			z	1358.5	-7.9	69.7	-0.7
3	1,203,019	183.3	x	595.5	437.5	9.9	26.9
			y	250.3	-0.6	162.8	-0.5
			z	1219.1	-7.6	-139.3	0.2
4	4,438,185	245.2	x	500.4	437.9	-95.2	0.4
			y	-22.1	0.1	-272.4	0.7
			z	1334.4	-5.3	115.3	2.3
5	6,114,845	219.0	x	502.3	441.8	1.9	3.9
			y	53.2	-0.1	75.3	-0.2
			z	1175.1	-4.5	-159.3	0.8
6	8,534,204	183.7	x	505.8	445.2	3.5	3.4
			y	67.8	-0.1	14.6	-0.1
			z	1223.0	-4.3	48.0	0.2
7	10,613,578	179.7	x	513.9	444.1	8.1	-1.1
			y	55.2	-0.1	-12.6	0.1
			z	1127.3	-3.8	-95.7	0.6

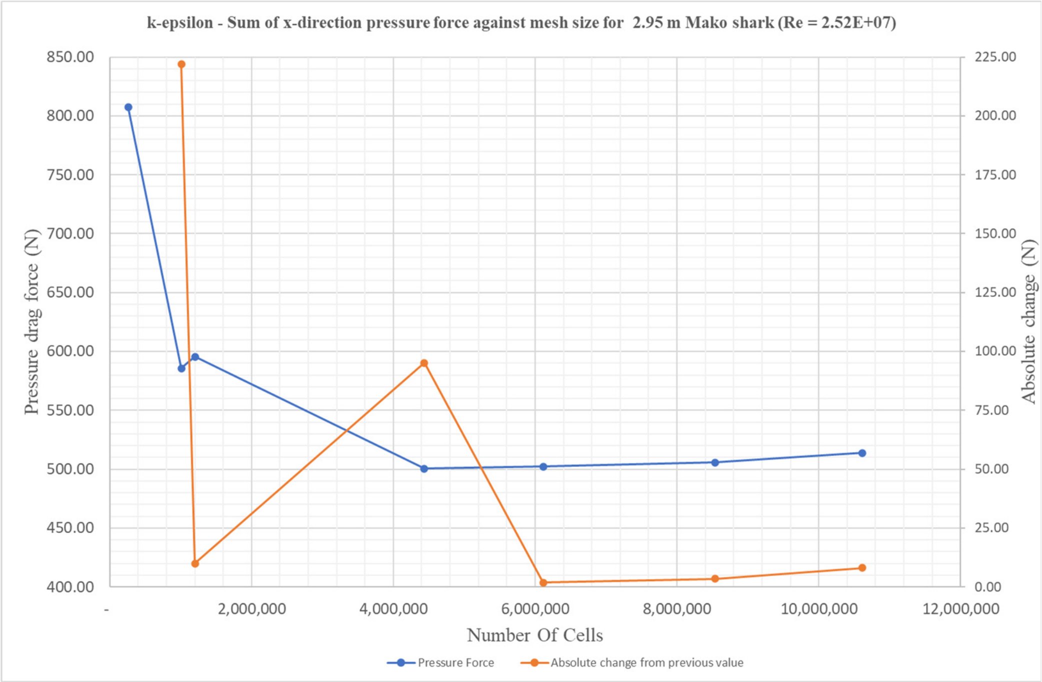

Figure S6. K-epsilon mesh independence results - pressure drag force. Changes in pressure drag force against mesh size for mesh 1-7 (primary axis), and the absolute change in this force from the previous value (secondary axis) (k-epsilon model)

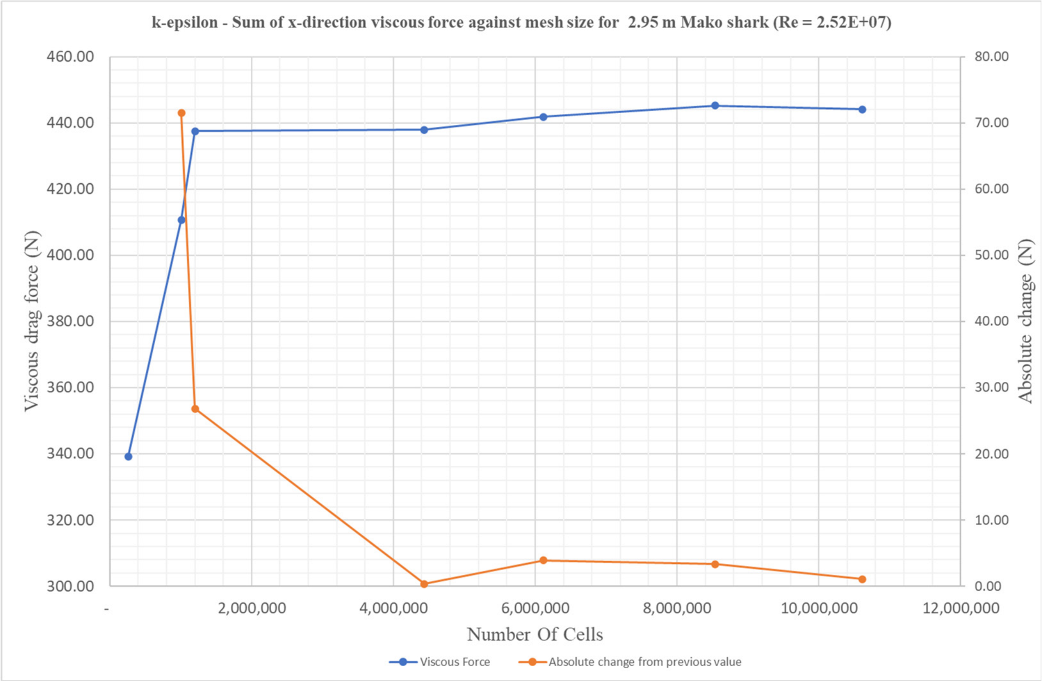

Figure S7. K-epsilon mesh independence results – viscous drag force. Showing a graph of the changes in viscous drag force against mesh size for mesh 1-7 (primary axis), and the absolute change in this force from the previous value (secondary axis) (k-epsilon model)

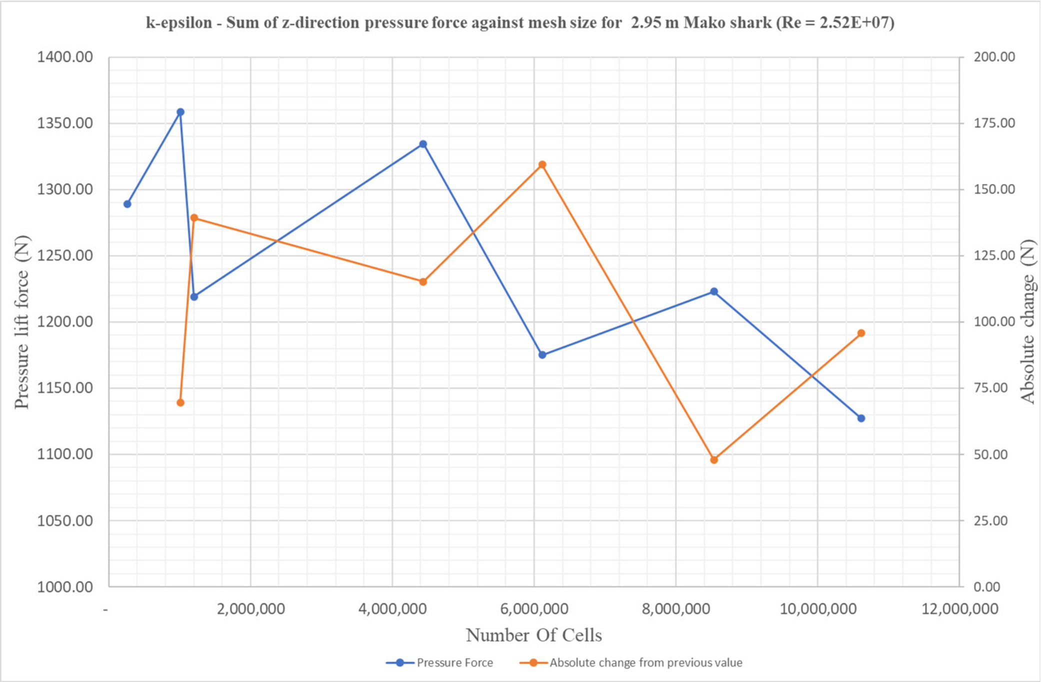

Figure S8. K-epsilon mesh independence results – lift force. Showing a graph of pressure force in the lift direction against the number of cells for the k-epsilon model; this force showed the least convergence, with a minimum relative change of 48.0 N.

B.2 k-omega SST results

Table S3. Results from the k-omega simulations for mesh 1-7.

Mesh	Average Y+ at walls	Direction	Sum of pressure forces [x y z] (N)	Sum of viscous forces [x y z] (N)	Relative change from previous value: pressure (1 , :) [x y z], viscous (2 , :) [x y z]	
1	833.9	x	743.5	388.4	~	~
		y	-144.5	0.2	~	~
		z	1236.0	-8.6	~	~
2	562.1	x	410.0	417.5	-333.5	29.1
		y	101.3	-0.1	245.7	-0.2
		z	1280.0	-8.4	44.1	0.2
3	420.0	x	362.8	434.5	-47.2	17.0
		y	239.3	-1.0	138.1	-0.9
		z	1104.3	-7.4	-175.8	0.9
4	254.3	x	252.6	450.0	-110.2	15.5
		y	-22.9	0.1	-262.3	1.1
		z	947.5	-4.1	-156.8	3.4
5	227.3	x	244.5	452.5	-8.1	2.5
		y	81.6	0.0	104.6	-0.1
		z	697.9	-2.4	-249.6	1.7
6	191.4	x	238.8	456.6	-5.7	4.1
		y	-10.1	0.0	-91.7	0.0
		z	649.4	-0.3	-48.5	2.1
7	187.7	x	234.9	457.2	-3.9	0.6

		y	-41.4	0.1	-31.3	0.1
		z	780.2	-3.2	130.8	-2.9

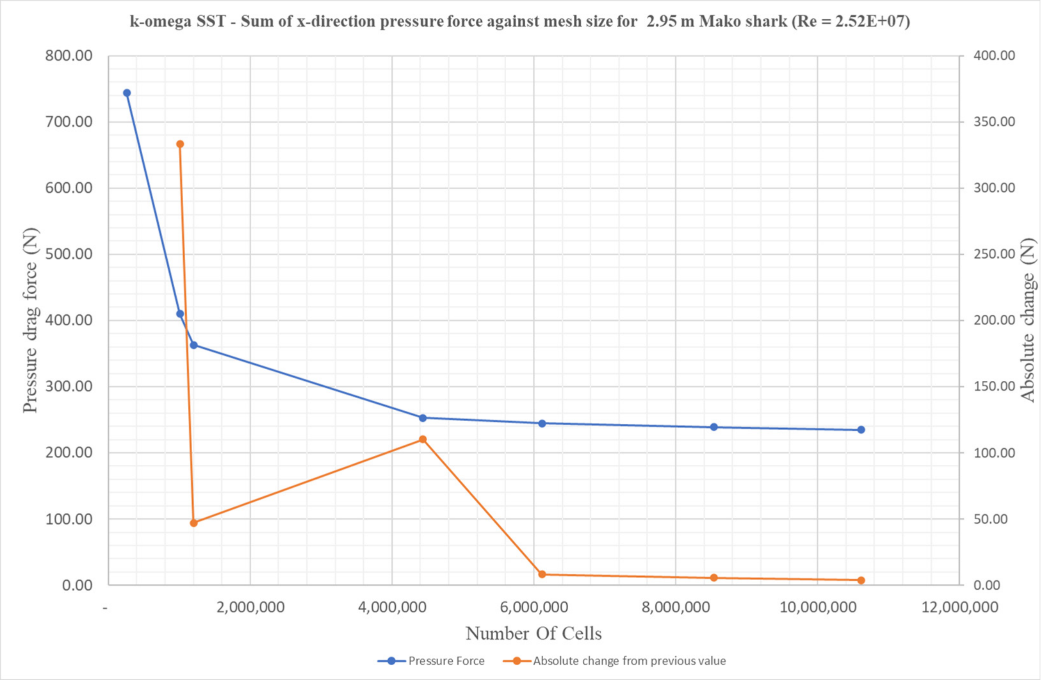

Figure S9. K-omega mesh independence results - pressure drag force. Showing a graph of the changes in pressure drag force against mesh size for mesh 1-7 (primary axis), and the absolute change in this force from the previous value (secondary axis) (k-omega SST model)

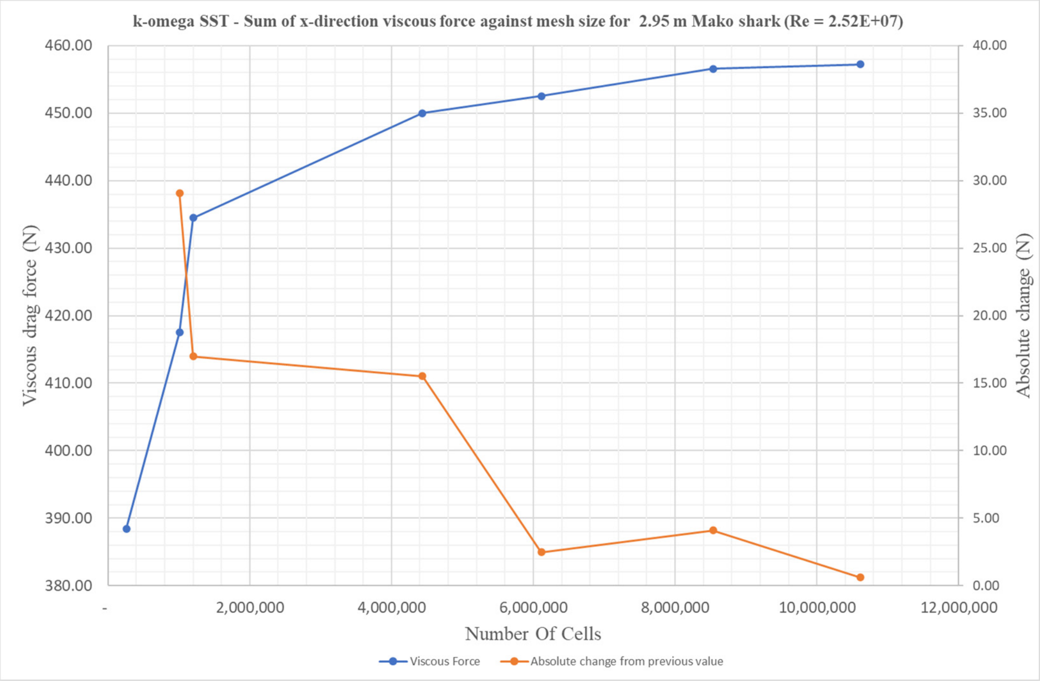

Figure S10. K-omega mesh independence results – viscous drag force. Showing a graph of the changes in viscous drag force against mesh size for mesh 1-7 (primary axis), and the absolute change in this force from the previous value (secondary axis) (k-omega SST model)

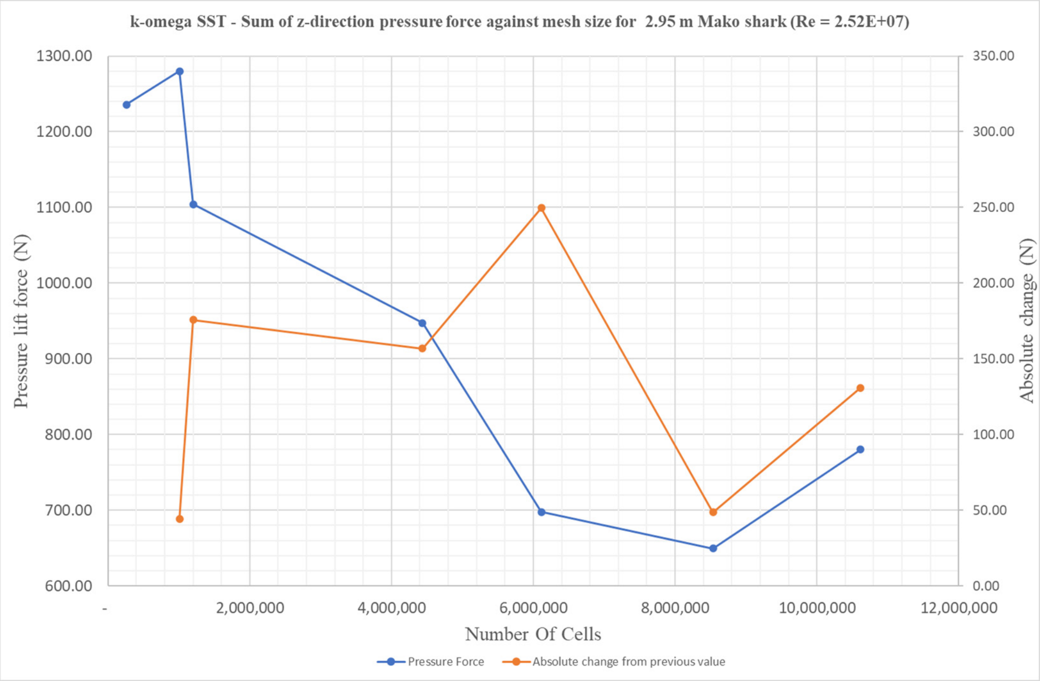

Figure S11. K-omega mesh independence results – lift force. Showing a graph of pressure force in the lift direction against the number of cells for the k-omega SST model; highlighting the force which was the least stable for variation in mesh size (minimum relative change of 48.5 N).

Supplementary C. Validation study using DARPA SUBOFF geometry and experimental tow tank data

A validation study was conducted to verify the accuracy of the CFD simulations against experimental data. The validation case used was the DARPA SUBOFF submarine model with tow tank test results (Groves et al., 1989; Huang and Liu, 1988). The DARPA SUBOFF geometry is a 1:24 linear scaled model of a submarine-like body with a length of L = 4.356 m and a maximum diameter of 0.508 m (Groves et al., 1989), which has been widely used as a benchmark case for CFD validation studies (Asad et al., 2023; Sezen et al., 2018; Bhushan et al., 2013). The study was selected for validation as it shares similar geometric characteristics with the mako shark geometry, such as a long-streamlined body with a similar characteristic length and maximum cross-sectional area. Furthermore, the flow velocities that are tested have a similar Reynolds number range as for the mako shark. The tow tank test results provide the net drag force on the DARPA SUBOFF (Huang and Liu., 1988), of which four data points were selected due to being closest to the range of this study which are given in Table S4.

Table S4. Results from the flow tank test of the DARPA SUBOFF geometry.

Reynold's number	Free Stream Velocity (m/s)	Experimental drag force (N)
$1.33{\times}10^{7}$	3.05	8.74E+01
$2.24{\times}10^{7}$	5.14	2.42E+02
$2.65{\times}10^{7}$	6.10	3.33E+02
$3.11{\times}10^{7}$	7.16	4.52E+02

Recreated from (Huang and Liu., 1988)

The geometric equations for creating the axisymmetric body (bow, parallel middle body, afterbody, and afterbody cap) are provided in Huang & Liu (1988); from this a MATLAB code was created to recreate the 2d outline by sampling the values of these equations along the length of the body, as shown in Figure S12.

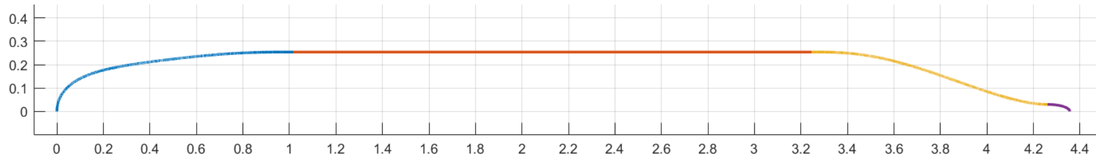

Figure S12 DARPA SUBOFF geometry created in MATLAB.

The code also functions to export the x, y, z coordinates of each section to separate text files, from which the 3d geometry was created in Solidworks. The sketch was created using the "Curve through XYZ points" feature to import the coordinates as a spline, and then using a revolved boss/base to create the 3d axisymmetric body, which was exported as an STL file using the finest resolution settings.

The DARPA SUBOFF geometry was then imported into a clean OpenFOAM case folder, with the same numerical and mesh settings used as for the mesh independent solutions. The study uses fresh water, which is assumed to be at 20°C, and so the properties are taken to be $\rho = 998.19\ kg/m^3$, and $\mu = 0.0010005\ N.s/m^2$ (The Engineering ToolBox, 2004; The Engineering ToolBox, 2003). The free stream turbulence intensity is given as 0.5% (Huang and Liu., 1988), from which the initial/boundary conditions were calculated for each flow velocity.

The meshing script was implemented to automate the meshing process, and a new script was created to automate the process of running multiple velocity simulations for the same geometry.

The CFD simulations were run for each Reynolds number using the simpleFoam solver with k-epsilon and k-OmegaSST turbulence models. The total drag force on the DARPA SUBOFF geometry was calculated from the sum of the x-direction pressure and viscous forces on its surface. The drag force values were compared with the experimental data to determine the absolute and percentage errors; in addition, the drag force was plotted against Reynolds number for the experimental, k-ε, and k- ω SST results; these metrics were used to validate and give confidence to the computational domain and CFD workflow, and to inform the selection of turbulence model chosen for the subsequent tag studies.

Table S5 shows the net drag force obtained from the CFD simulations using the k-epsilon and k-omega SST turbulence models, as well as the experimental drag force from Huang & Liu (1988). Table S6 shows the error and percentage error between the experimental and simulated drag force for both turbulence models. Figure S13 shows the comparison of the net drag force between the experimental and simulated drag force over the range of Reynolds number flows tested.

Table S5. Results of k-epsilon and k-omega SST models and experimental drag force from the DARPA SUBOFF tow tank tests in (Huang and Liu, 1988).

			Simulated drag force [Sum of pressure and viscous drag in x-direction] (N)	
Reynold's number	Free Stream Velocity (m/s)	Experimental drag force (N)	k-epsilon - Mesh 5	k-omega SST - Mesh 6
1.33×10^{7}	3.05	87.40	92.95	91.33
2.24×10^{7}	5.14	242.20	289.45	242.65
2.65×10^{7}	6.10	332.90	393.22	332.53
3.11×10^{7}	7.16	451.50	537.30	448.24

Table S6. Error between experimental and simulated drag force for the k-epsilon and k-omega SST models.

Error (N)		Percentage Error (%) [$= \mathbf{100} * \left(\frac{error}{experimental\ value}\right)$]	
k-epsilon	k-omega SST	k-epsilon	k-omega SST
5.55	3.93	6.35	4.50
47.25	0.45	19.51	0.19
60.32	-0.37	18.12	-0.11
85.80	-3.26	19.00	-0.72

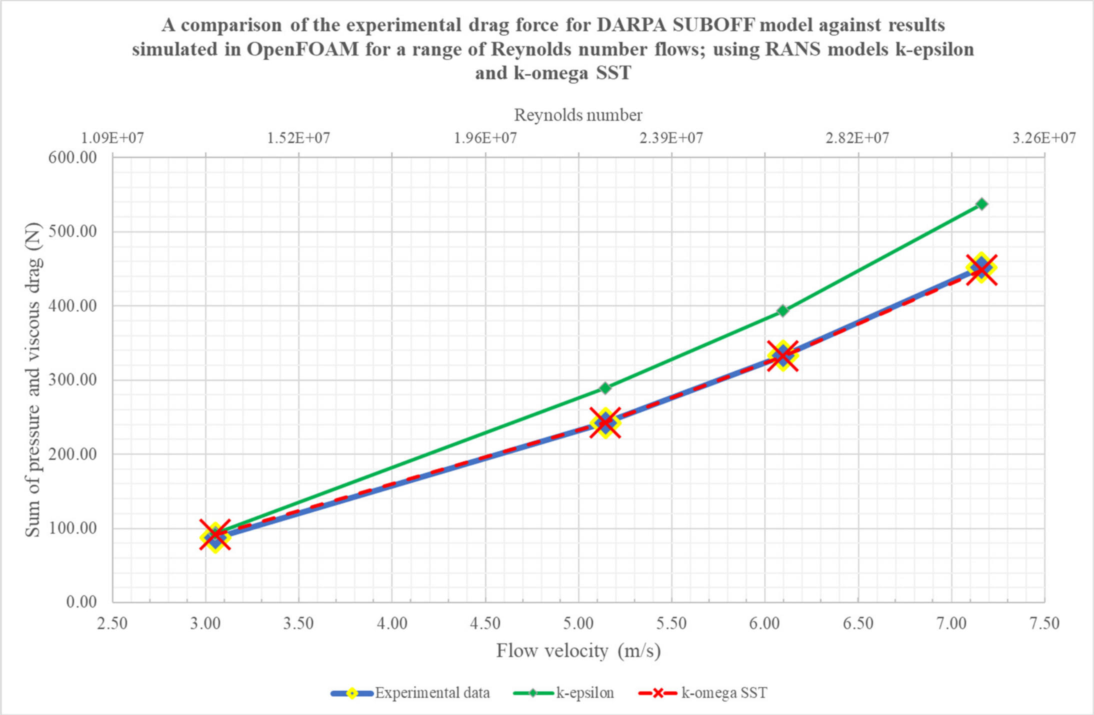

Figure S13. DARPA-SUBOFF model validation. Showing a comparison of the experimental and simulated drag force over the range of Reynolds number flows tested; highlighting the benefits of the k-omega SST model for this case, with a resulting error of less than 4N for all simulation.

The results show that the k-epsilon model consistently overpredicts the net drag force compared to the experimental data for all Reynolds numbers, as the error values are positive and large for all cases, ranging from 5.55 N to 85.80 N. The k-omega SST model, on the other hand, predicts the net drag force more accurately than the k-epsilon model, as the error values are small and appear to fluctuate around the true value, ranging from -3.26 N to 3.93 N. The k-omega SST model has a consistently lower percentage error than the k-epsilon model for all cases, ranging from -0.72% to 4.50%, while for the k-epsilon model this is 6.35% to 19.51%. Overall, the net drag force values for both turbulence models follow the trend of the experimental data, however the k-omega SST model shows much better agreement with the experimental results for the range of Reynolds numbers tested, with all values within 4N of the experimental values; this provides confidence in the computational domain, choice of mesh resolution and overall CFD methodology.

As a result, the k-omega SST model is selected for use as the main turbulence model for the subsequent tag studies. However, a limitation of this validation study is that the DARPA SUBOFF geometry is simpler than the shark geometry, as it lacks the appendages (fins etc.) and curvature variation along the body; as shown in Figure S14, this creates more complex flow characteristics, meaning that the error of the results for the shark geometry may be larger than the error of the results for the DARPA SUBOFF geometry.

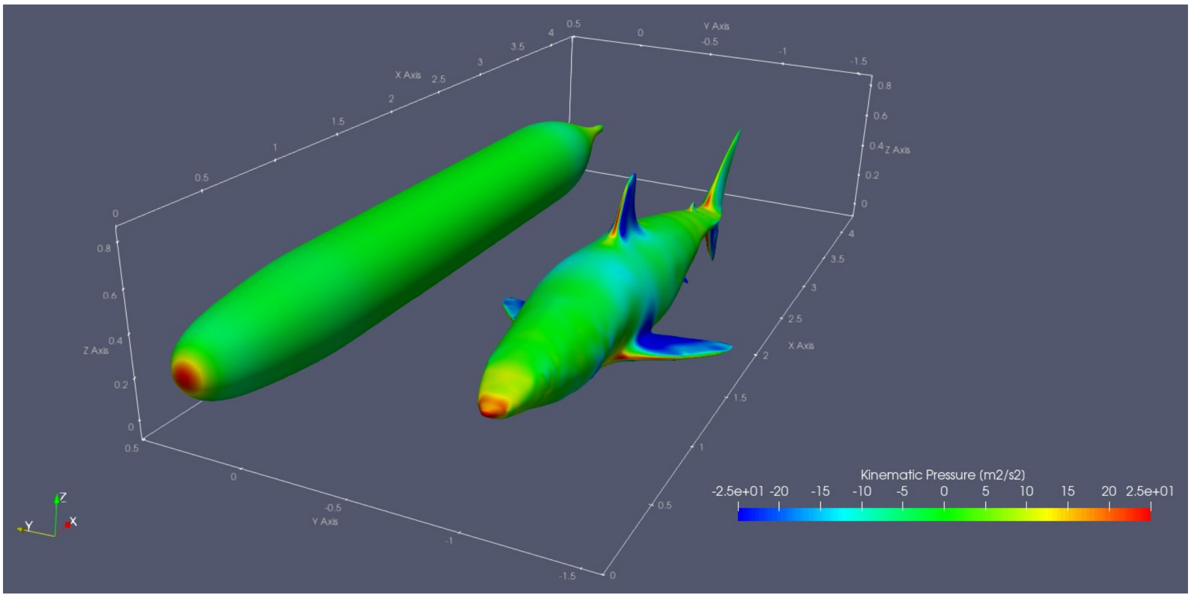

Figure S14. Comparison of the surface pressure distribution of the Mako shark and DARPA SUBOFF geometry.

Supplementary D. Results of modelling case 1 – effects of MiniPAT and SPOT tag on full-size shark model

Table S7. Dimensionless coefficients for an untagged Mako shark.

Average y+ at walls	Reynolds number	Free Stream Velocity (m/s)	C_D	C_L	C_M	Convergence
10.0	1.39×10^6	0.50	0.121	-0.139	-0.025	1.20×10^{-3}
24.6	2.77×10^6	1.00	0.098	-0.060	-0.022	9.00×10^{-4}
37.1	4.16×10^6	1.50	0.089	-0.024	-0.021	6.70×10^{-4}
48.5	5.55×10^6	2.00	0.084	-0.002	-0.020	6.60×10^{-4}
82.4	9.99×10^6	3.60	0.074	0.038	-0.019	5.50×10^{-4}
115.1	1.39×10^7	5.00	0.071	0.074	-0.013	5.50×10^{-4}
172.4	2.16×10^7	7.80	0.067	0.076	-0.012	5.50×10^{-4}
198.2	2.52×10^7	9.10	0.066	0.076	-0.012	5.50×10^{-4}

Table S8. Dimensionless coefficients of a Mako shark with a MiniPAT tag (placement 1 and 2) for a range of test velocities.

	MiniPAT Placement 1				MiniPAT Placement 2			
Reynolds number	C_D	C_L	C_M	Convergence	C_D	C_L	C_M	Convergence
1.39E+06	0.123	-0.142	-0.024	8.90×10^{-4}	0.123	0.141	0.024	1.40×10^{-3}
2.77E+06	0.099	-0.068	-0.022	8.80×10^{-4}	0.099	0.066	0.022	8.00×10^{-4}
4.16E+06	0.091	-0.033	-0.021	7.70×10^{-4}	0.091	0.026	0.019	6.90×10^{-4}
5.55E+06	0.086	-0.012	-0.020	6.60×10^{-4}	0.086	0.006	0.019	8.00×10^{-4}
9.99E+06	0.074	0.017	-0.020	5.50×10^{-4}	0.077	0.026	0.019	5.50×10^{-4}
1.39E+07	0.073	0.037	-0.011	5.50×10^{-4}	0.073	0.047	0.019	5.50×10^{-4}
2.16E+07	0.065	0.078	-0.012	5.50×10^{-4}	0.065	0.071	0.015	5.50×10^{-4}
2.52E+07	0.064	0.079	-0.015	5.50×10^{-4}	0.064	0.075	0.015	5.50×10^{-4}

Table S9. Dimensionless coefficients of a Mako shark with a MiniPAT tag (placement 3 and 4) for a range of test velocities.

	MiniPAT Placement 3				MiniPAT Placement 4			
Reynolds number	C_D	C_L	C_M	Convergence	C_D	C_L	C_M	Convergence
1.39E+06	0.123	-0.141	-0.024	8.90×10^{-4}	0.123	0.141	0.024	1.40×10^{-3}
2.77E+06	0.098	-0.068	-0.022	8.80×10^{-4}	0.098	0.068	0.022	8.00×10^{-4}
4.16E+06	0.090	-0.024	-0.021	7.70×10^{-4}	0.091	0.024	0.021	6.90×10^{-4}
5.55E+06	0.085	-0.004	-0.020	6.60×10^{-4}	0.086	0.004	0.020	8.00×10^{-4}

9.99E+06	0.078	0.049	-0.020	5.50×10^{-4}	0.073	0.049	0.020	5.50×10^{-4}
1.39E+07	0.073	0.067	-0.011	5.50×10^{-4}	0.072	0.067	0.011	5.50×10^{-4}
2.16E+07	0.066	0.073	-0.012	5.50×10^{-4}	0.069	0.073	0.012	5.50×10^{-4}

Table S10. Dimensionless coefficients of a Mako shark with a dorsal fin SPOT tag for a range of test velocities.

SPOT tag (Dorsal Fin)				
Reynolds number	C_D	C_L	C_M	Convergence
1.39E+06	0.143	-0.141	-0.023	1.30×10^{-3}
2.77E+06	0.118	-0.070	-0.022	7.00×10^{-4}
4.16E+06	0.109	-0.026	-0.020	6.00×10^{-4}
5.55E+06	0.104	-0.009	-0.018	6.00×10^{-4}
9.99E+06	0.096	0.018	-0.017	7.00×10^{-4}
1.39E+07	0.092	0.031	-0.016	8.40×10^{-4}
2.16E+07	0.086	0.054	-0.015	7.60×10^{-4}
2.52E+07	0.084	0.084	-0.014	1.00×10^{-4}

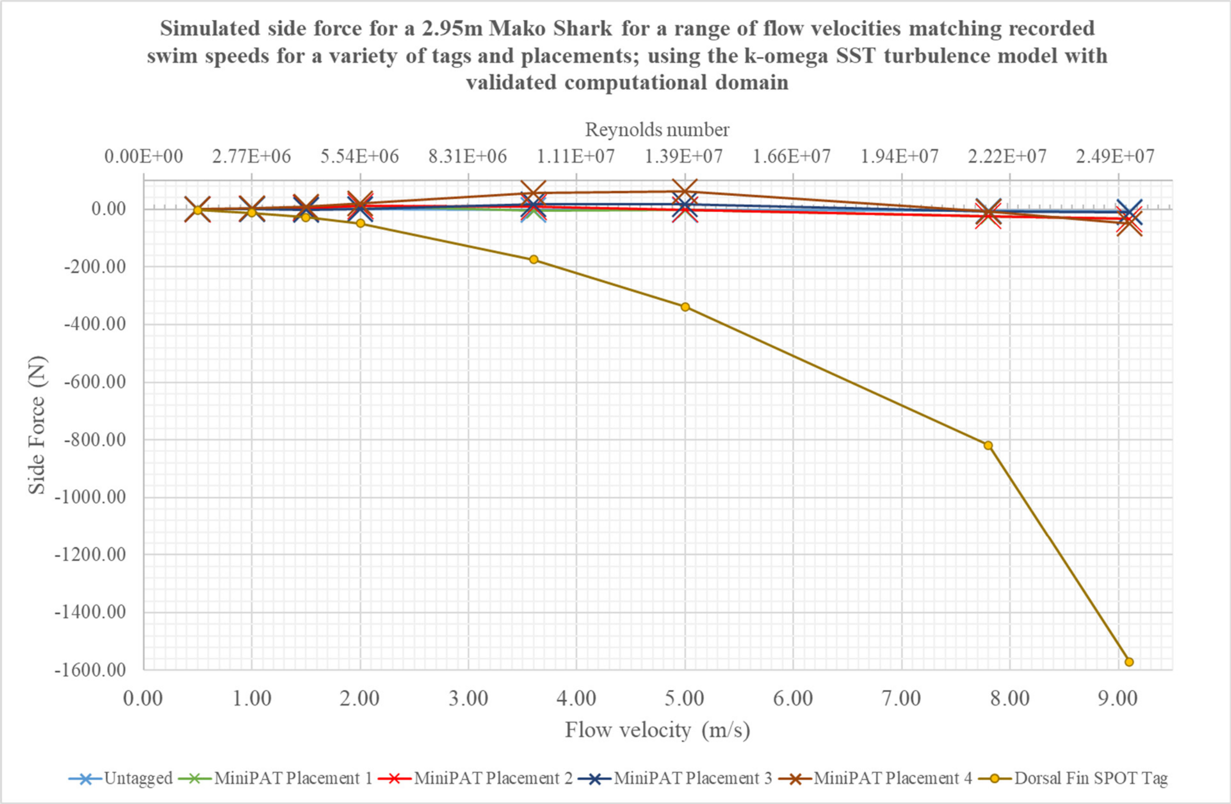

Figure S15. Simulated side force acting on tagged and untagged Mako shark for the range of flow velocities tested.

Supplementary E. Results of modelling case 2 – effects of MiniPAT tags on forces acting on mako shark models of varying sizes

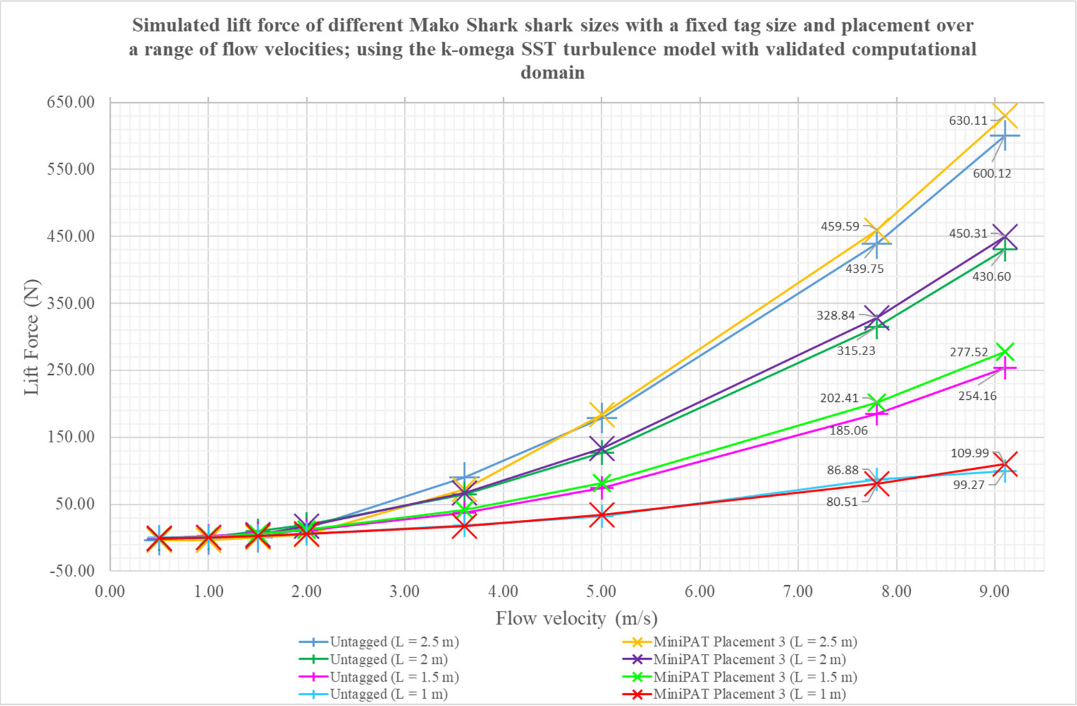

Figure S16. Lift force acting on the tagged and untagged Mako shark for a range of shark sizes.

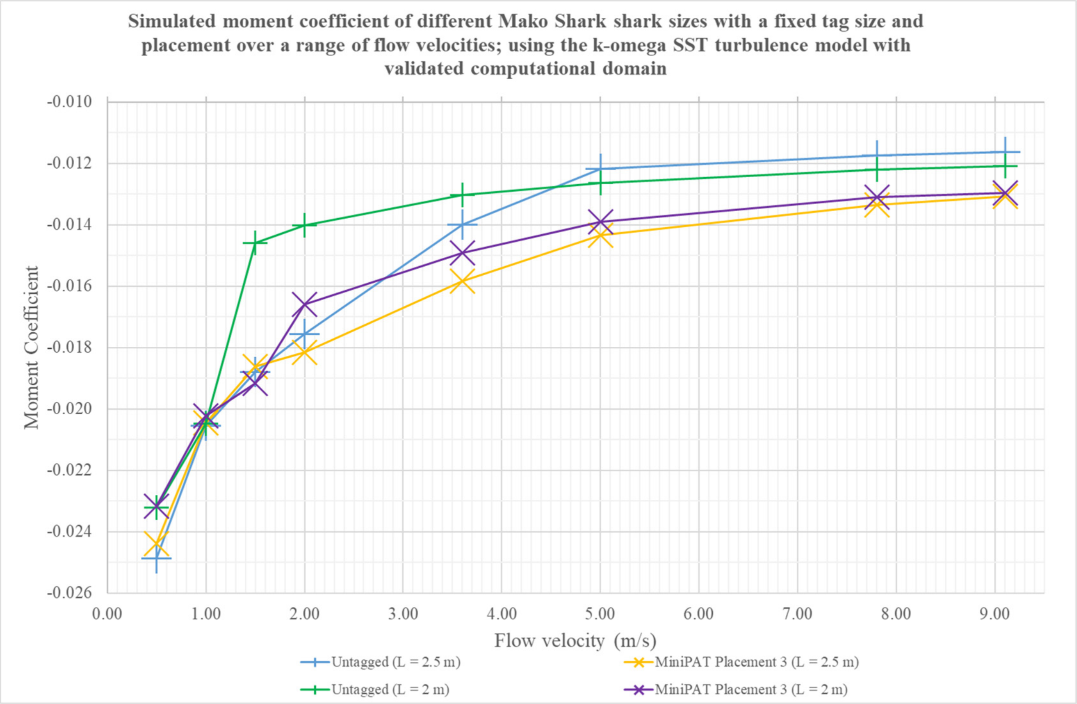

Figure S17. Pitching moment coefficient for the untagged and tagged cases of L = 2.5 m and 2 m.

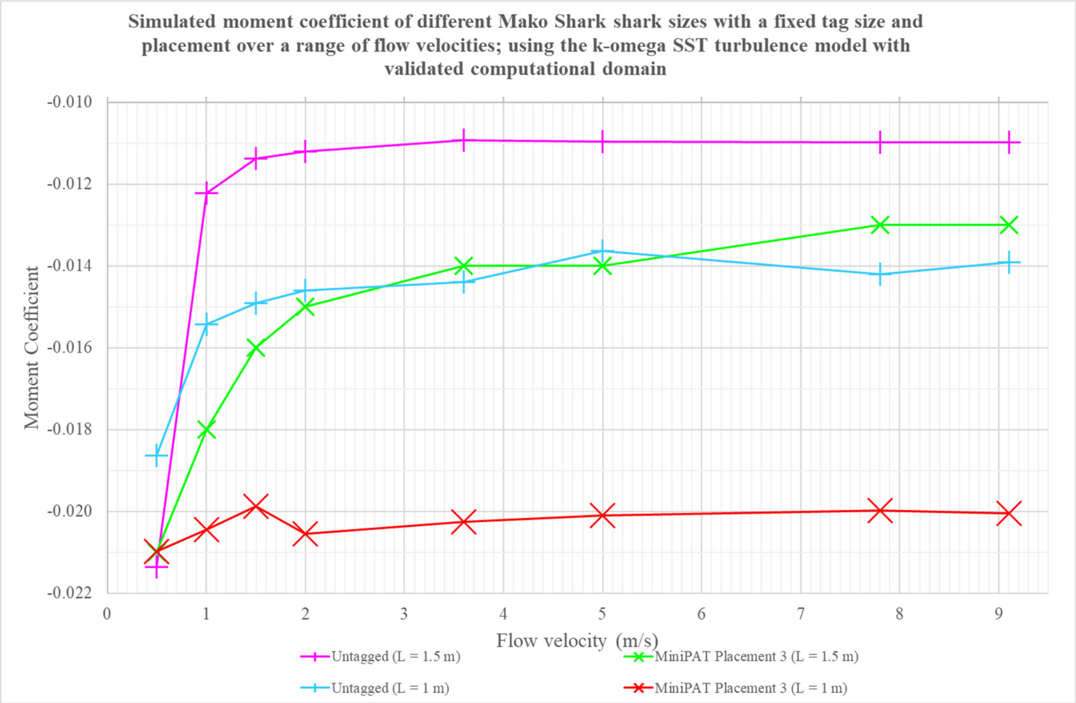

Figure S18. Pitching moment coefficient for the untagged and tagged cases of L = 1.5 m and 1 m.

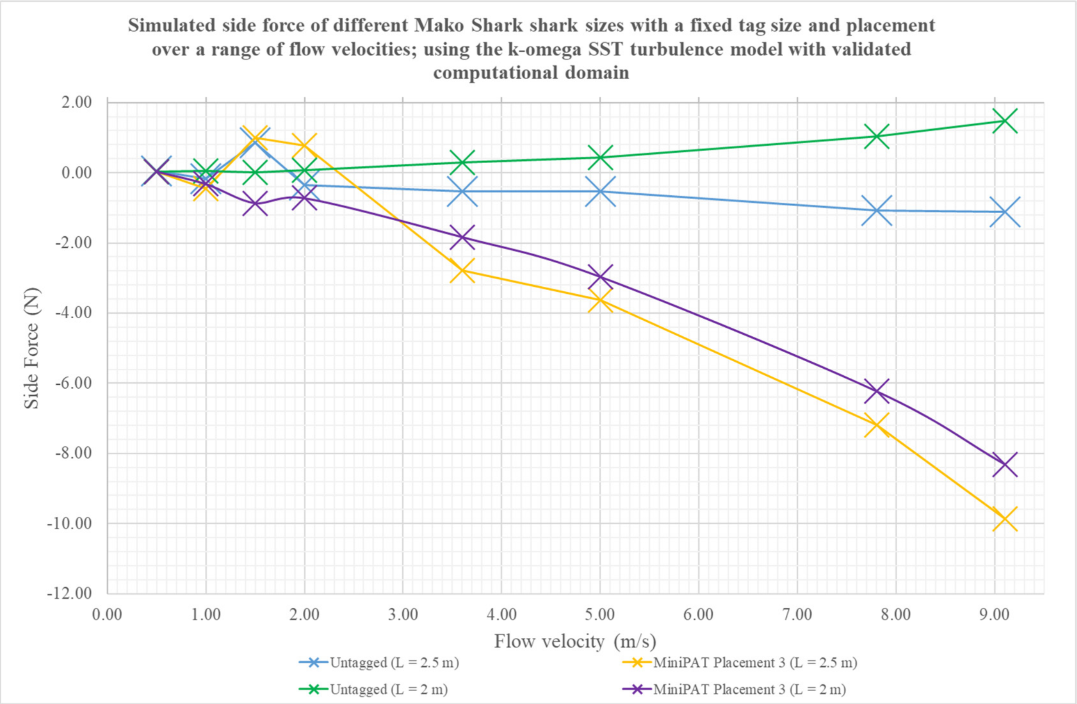

Figure S19. Side force for the untagged and tagged cases of L = 2.5 m and 2 m.

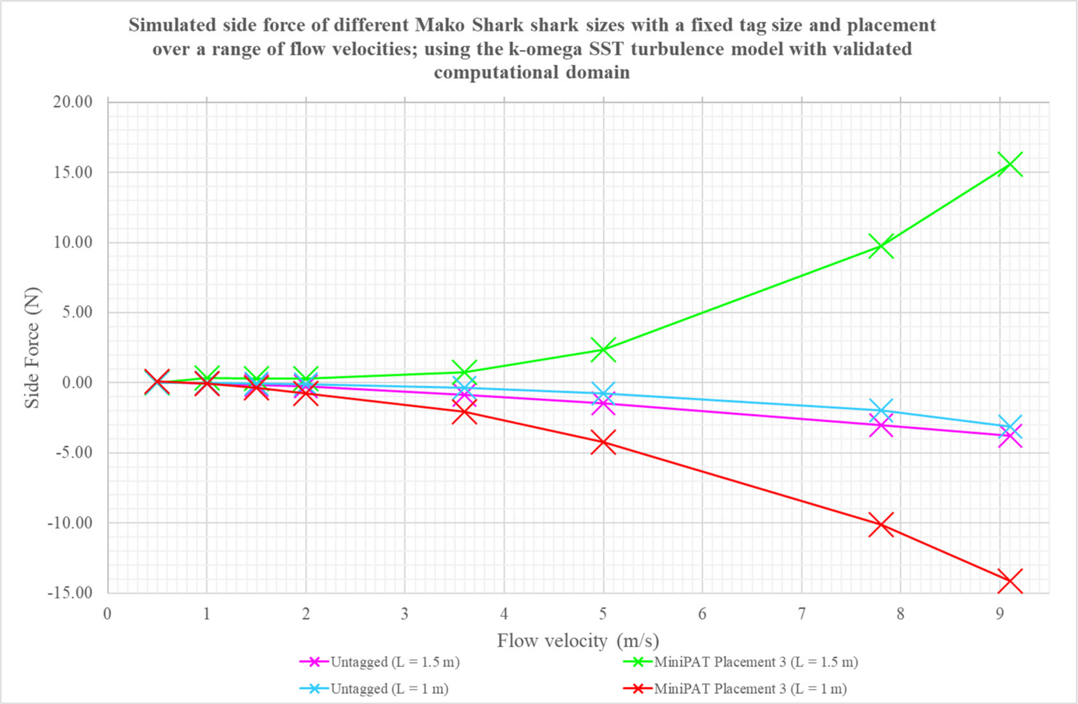

Figure S20. Showing a plot of side force against flow velocity for the untagged and tagged cases of L = 1.5 m and 1 m.
