# Supplementary material for: Refining Electronic Tagging of Marine Animals: Computational Fluid Dynamics and Pelagic Sharks
Source: Animals (Basel). 2025 Oct 13;15(20):2956. doi: 10.3390/ani15202956 (PMC12560902; doi:10.3390/ani15202956)

Refining electronic tagging of marine animals: computational fluid dynamics and pelagic sharks

Tobias Maillard, Francesco Garzon, Lucy A. Hawkes, Gavin Tabor and Matthew J. Witt

Supplementary Material S2 - Example look-up table for ethical appraisal of shark tagging studies.

The table in example offers a simple, if unrefined, method of assessing the likely cost of the chosen tag type on the species of interest.
A researcher could find the table most relevant to the shark species of interest to the project, based on its general body plan, and immediately estimate the potential energetic cost range incurred by the animal as function of its swimming speed.

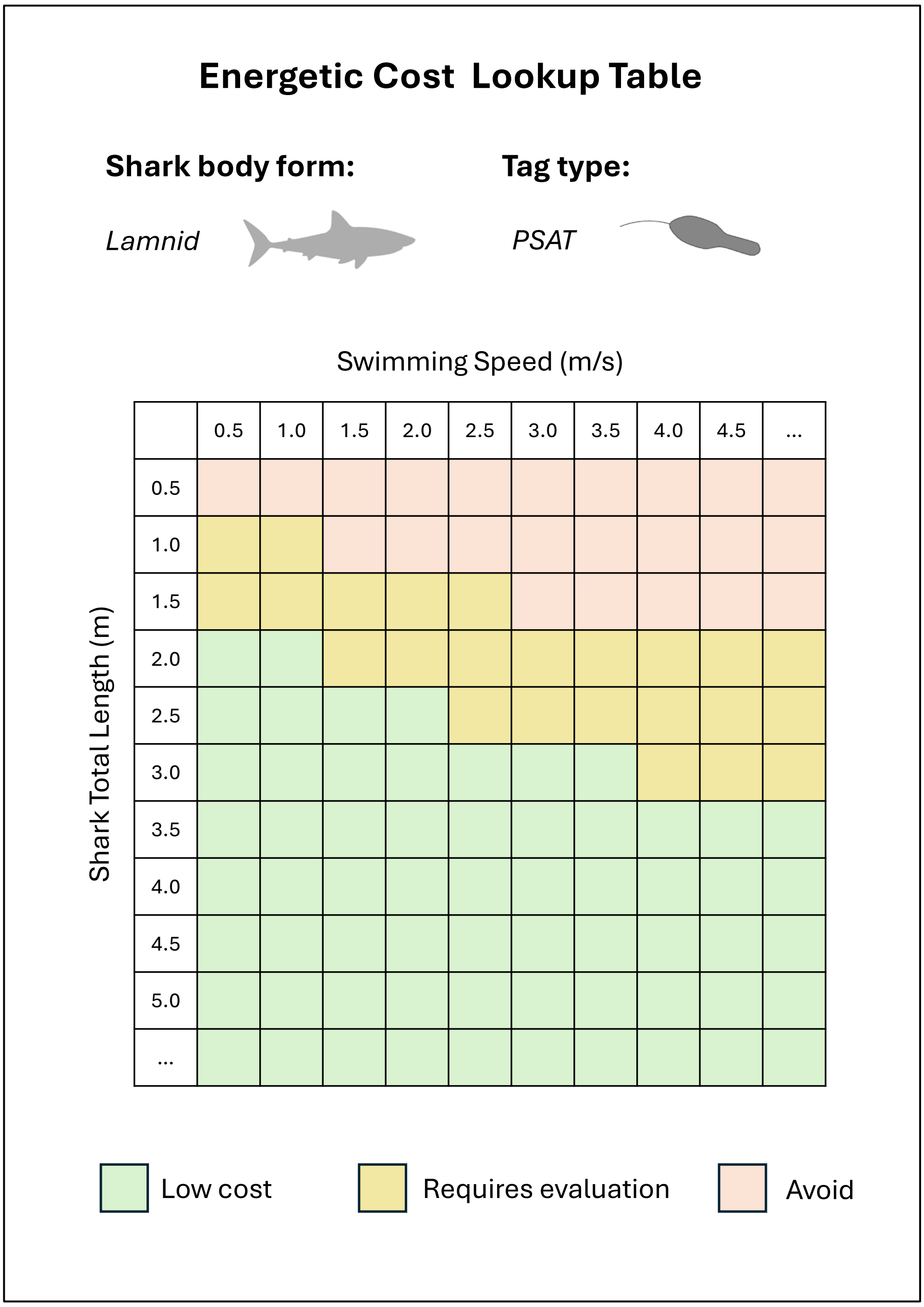

Supplement: Supplementary file 1 [file animals-15-02956-s001.zip › FileS2_LookupTable.pdf]
